# Interactions of Graphene Oxide and Few-Layer Graphene with the Blood–Brain Barrier

**DOI:** 10.1021/acs.nanolett.3c00377

**Published:** 2023-03-14

**Authors:** Valentina Castagnola, Lieselot Deleye, Alice Podestà, Edra Jaho, Fabrizio Loiacono, Doriana Debellis, Martina Trevisani, Dinu Zinovie Ciobanu, Andrea Armirotti, Francesco Pisani, Emmanuel Flahaut, Ester Vazquez, Mattia Bramini, Fabrizia Cesca, Fabio Benfenati

**Affiliations:** † Center for Synaptic Neuroscience and Technology, Istituto Italiano di Tecnologia, Largo Rosanna Benzi 10, 16132 Genova, Italy; ‡ IRCCS Ospedale Policlinico San Martino, Largo Rosanna Benzi 10, 16132 Genova, Italy; § Electron Microscopy Facility, Istituto Italiano di Tecnologia, Via Morego, 30, 16163 Genova, Italy; ∥ Analytical Chemistry Lab, Istituto Italiano di Tecnologia, Via Morego 30, 16163 Genova, Italy; ⊥ Department of Biosciences, Biotechnologies and Biopharmaceutics, University of Bari “Aldo Moro”, Bari 70121, Italy; # CIRIMAT, UMR 5085, CNRS-INP-UPS, Université Toulouse 3 Paul Sabatier, 118 route de Narbonne, F-31062 Toulouse cedex 9, France; ∇ Instituto Regional de Investigación Científica Aplicada (IRICA), Universidad de Castilla-La Mancha, 13071 Ciudad Real, Spain; ○ Facultad de Ciencias y Tecnologías Químicas, Universidad de Castilla-La Mancha, Avda. Camilo José Cela S/N, 13071 Ciudad Real, Spain; ● Department of Cell Biology, Universidad de Granada, C. Fuentenueva s/n, 18071 Granada, Spain; ▲ Department of Life Sciences, University of Trieste, 34127, Trieste, Italy; @ Department of Experimental Medicine, Università degli Studi di Genova, 16132 Genova, Italy

**Keywords:** graphene, blood−brain barrier, assembloids, uptake pathways, tight junctions

## Abstract

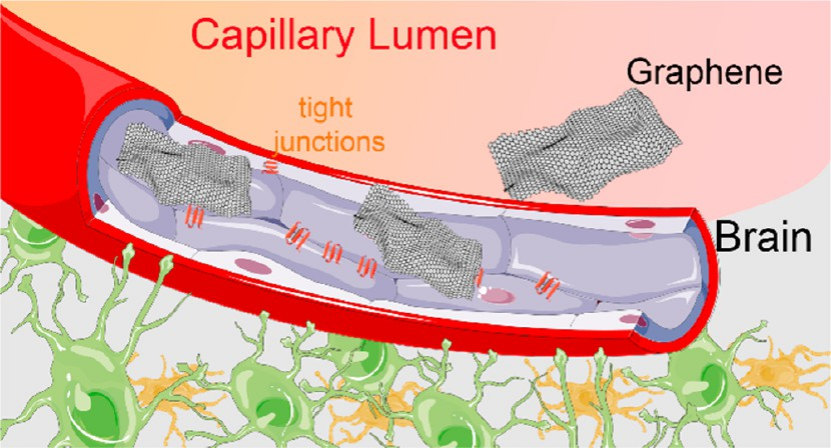

Thanks to their biocompatibility and high cargo capability, graphene-based materials (GRMs) might represent an ideal brain delivery system. The capability of GRMs to reach the brain has mainly been investigated *in vivo* and has highlighted some controversy. Herein, we employed two *in vitro* BBB models of increasing complexity to investigate the bionano interactions with graphene oxide (GO) and few-layer graphene (FLG): a 2D murine Transwell model, followed by a 3D human multicellular assembloid, to mimic the complexity of the *in vivo* architecture and intercellular crosstalk. We developed specific methodologies to assess the translocation of GO and FLG in a label-free fashion and a platform applicable to any nanomaterial. Overall, our results show good biocompatibility of the two GRMs, which did not impact the integrity and functionality of the barrier. Sufficiently dispersed subpopulations of GO and FLG were actively uptaken by endothelial cells; however, the translocation was identified as a rare event.

Graphene, in the form of either a colloidal suspension or a planar substrate, has been considered an exciting biomaterial for biological applications, and its interactions with the central nervous system (CNS) have been widely investigated in the past decade.^1−6^

Like all other nano/micro materials, graphene-based materials, when administered systemically, must cross the blood–brain barrier (BBB) in order to access the brain. The BBB is an essential regulatory layer at the neural interface with the brain vasculature, which acts as a selective barrier. The tight junctions forming between adjacent cells prevent molecules from moving paracellularly, forcing them to take a transcellular route for their translocation.^7,8^ The tightly controlled chemical composition of the extracellular *milieu* of the CNS, provided by the barrier, is essential for correct neural functioning; indeed, several diseases are associated with the BBB local disruptions.^9,10^ However, the presence of the BBB also hinders the delivery of therapeutics to the brain, and therefore the clinical success in overcoming the BBB for therapeutic needs has been very limited when using molecular approaches.^11−13^

The idea of exploiting nanomaterials to overcome the BBB has attracted growing interest in the past decade.^14−19^ In the nanosize range, the engagement with the biological membranes allows for active transport mechanisms of internalization and transcytosis that are compatible with the process of BBB translocation. Although this phenomenon is often observed as a rare event,^20−22^ nanomaterials offer a plethora of opportunities that might allow boosting the BBB crossing, such as surface chemistry engineering, downsizing, and hybrid constructs decorated with endogenous motifs.^23−28^

In this context, colloidal graphene-based materials (GRMs), with their proven biocompatibility and excellent cargo capability, are considered very promising.^29−33^

Several reports so far have investigated the biodistribution of various colloidal GRMs injected systemically, suggesting that it is unlikely for GRMs to cross the BBB and accumulate in the brain.^34−38^ Despite a certain consensus, some controversial results can be found across the literature, reporting BBB transient disruption or brain accumulation.^39,40^ However, to date, detailed knowledge of the molecular interactions between GRMs and BBB cells and architecture is missing.^41^

In this work, we performed an *in vitro* investigation of two GRM interactions with the BBB. To this aim, we employed two GRMs with different surface chemistry and stability, both murine and human models of increasing complexity, and a portfolio of complementary label-free analytical techniques. The use of label-free strategies allows the avoidance of fluorescent tag interference and leaching, which makes the results of the bionano interactions hard to interpret.^42,43^ The workflow developed in this paper is widely applicable to the study of the translocation of different micro- and nanomaterials and represents a valuable method to reduce the more expensive and ethically concerning *in vivo* biodistribution studies.

## Graphene Interactions with a 2D Murine Model of BBB

For this study, two main graphene-based materials (GRMs) were employed: few-layer graphene (FLG) and graphene oxide (GO). These materials present similar sizes and morphological features but different surface chemistry, and their characterization is extensively described in our previous works and also reported in Figure S1.^44,45^ Reduced graphene oxide (RGO) was also employed for some initial investigations. RGO material is obtained from GO, reduced at high temperatures (800 °C), as described elsewhere (characterization is shown in Figure S2).^46^ The surface chemistry of RGO should then be more similar to FLG, but it can still present oxygen defects on the surface. As shown in the Turbiscan analysis of Figure S3, the colloidal stability of RGO in the biological environment over time was very poor compared to that of FLG and GO.

The first BBB model that we employed was a monolayer culture of murine brain endothelial cells (bEnd.3). We initially checked whether GO and FLG had any detrimental effects on the barrier features, such as cell viability, morphology, tight junction expression, and barrier functionality. For imaging purposes, bEnd.3 cells were cultured on glass coverslips, while functional tests were performed using Transwell membranes (see Methods in the Supporting Information). Cells were incubated with 10 μg/mL of each GRM at different time points. No effects on cell viability, monolayer organization, cell morphology, or polarization were observed after 24 h of exposure, as reported in Figure S4.

**Figure 1:**
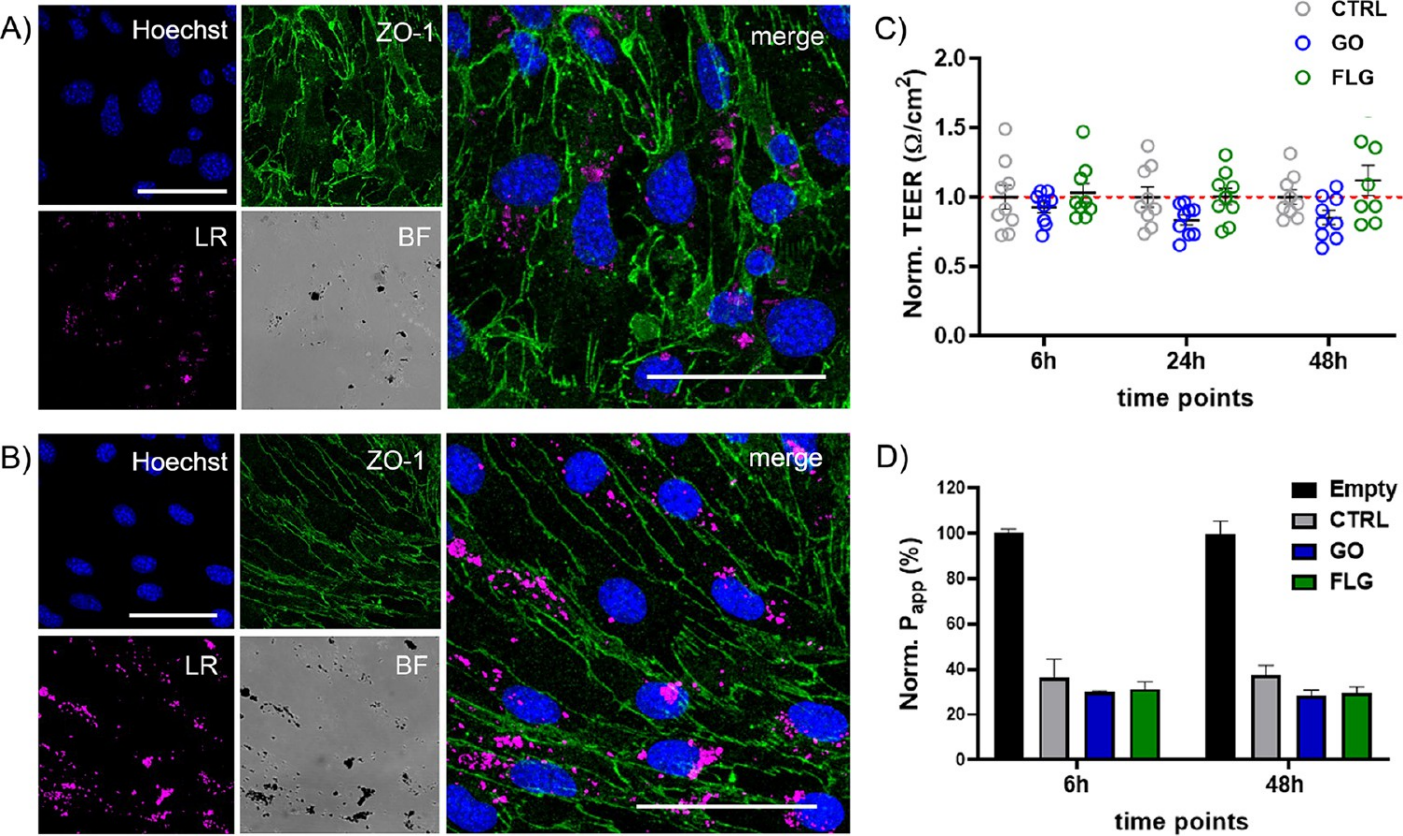
Characterization of the BBB properties upon GRM exposure. Representative confocal images of immunofluorescence staining for ZO-1 (green) in bEnd.3 monolayers seeded on glass coverslips after 48 h exposure to 10 μg/mL of GO (A) or FLG (B). The Hoechst dye was used for nuclei visualization (blue). GRM particles are visible in light reflection mode (LR) mode (pink). BF denotes bright field image. Scale bars: 50 μm. (C) TEER values after exposure to 10 μg/mL of GO and FLG for 6, 24, and 48 h. For each time point, values were normalized over controls and are represented as means ± SD (*n* = 9 independent preparations). *p* > 0.05, one-way ANOVA/Tukey’s tests. (D) Apparent permeability (*P*_app_, %) of FD4 after exposure to 10 μg/mL of GO and FLG for 6 and 48 h. *P*_app_ of a Transwell without cells (Empty) is indicated as 100%, and values are normalized accordingly. Values are represented as means ± SEM (*n* = 3).

Figure 1A,B shows confocal imaging of bEnd.3 cells exposed for 48 h to GO and FLG, respectively. The presence of GRM flakes in the cells can be visualized using the light reflection (LR) mode (in pink in the figure). The immunostaining for zonula occludens-1 (ZO-1), one of the main TJ proteins expressed in endothelial cells, shows a physiological expression of the protein localized at the cell membrane with no major alteration due to GO and FLG exposure. More confocal images are reported in Figure S5.

For bEnd.3 cells cultured on Transwell membranes, the barrier properties were assessed by measuring the trans-endothelial electrical resistance (TEER). As shown in Figure S6, TEER values for the cell monolayer stabilized around 10–15 Ω cm^2^ starting from day 6. Figure 1C depicts the TEER analyses performed after exposing the cells to 10 μg/mL of GO and FLG for 6, 24, and 48 h. The apparent permeability (*P*_app_) was also measured upon exposure to the two GRMs, using a fluorescent probe (dextran 4 kDa FITC labeled (FD4); Figure 1D). Overall, the GRM treatments did not significantly affect any of the observed barrier properties, confirming the integrity and functionality of the tight junctions. TEER and *P*_app_ results related to RGO are reported in Figure S7.

Previous studies reported the influence of pristine graphene and GO on signaling pathways and their role in inducing changes in the expression and regulation of genes and proteins in various mammalian cells.^47−50^ For this reason, following the exposure of bEnd.3 cells to either FLG or GO, we explored the cell proteome by obtaining label-free high-resolution LC-MS data on the cell lysates.

We quantified a total of 5153 protein groups in all three groups (control, FLG, and GO), with a total protein abundance profile that spans over roughly 4.5 logs. The full list of quantified proteins is reported in Supplementary File P1 and can also be found in the PRIDE database (data set identifier PXD038297). We detected only minor changes in the bEnd.3 proteome following exposure to GO (134 proteins) and FLG (43 proteins), corresponding to about 2.6% and 0.8% of the total observed bEnd.3 proteome, respectively. The volcano plots reported in Figure S8 show the proteins (red dots) that were significantly (*p* < 0.05) altered compared to the total quantified proteome (gray dots) for GO and FLG exposure, respectively. The full sets of altered proteins are reported in Supplementary Files P2 and P3. A subsequent gene enrichment analysis failed to highlight any cell process or function significantly altered by the exposure to GO or FLG.

## GRM Uptake in the 2D BBB Model

Once the persistence of the barrier functionality and the tightness of the paracellular spaces under our GRM exposure conditions were assessed, we then moved to investigate the capability of the two GRMs to cross the BBB through transcellular transport. The stability of the GRM dispersion plays a crucial role in the processes of internalization and translocation.

**Figure 2:**
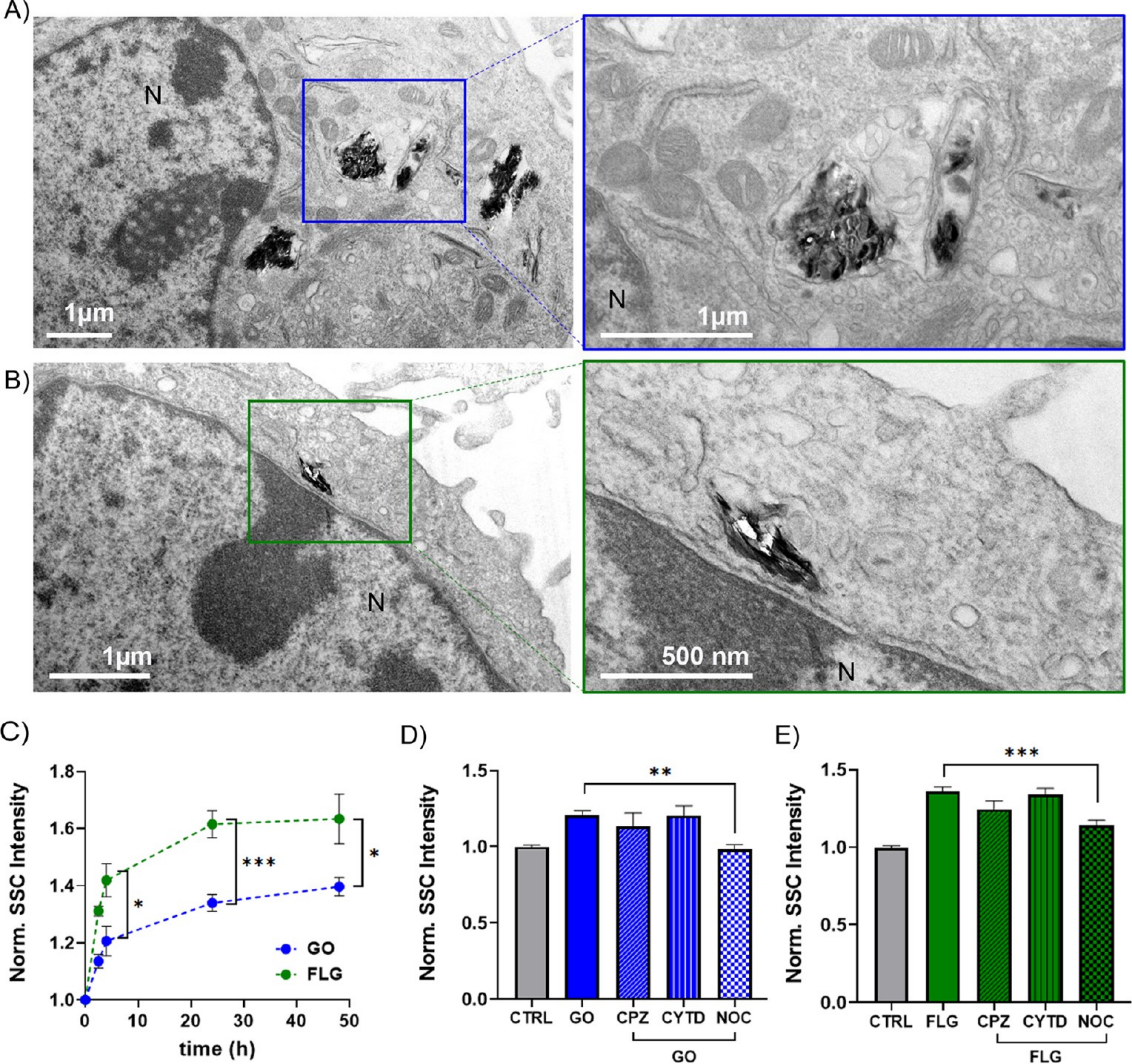
Uptake kinetics and internalization pathways of GO and FLG. Representative TEM micrographs showing cellular internalization of GO (A) and FLG (B) flakes in bEnd.3 cells. In the lower-magnification micrographs on the left, N indicates the nucleus. (C) Uptake kinetics for GO and FLG (10 μg/mL) in bEnd.3 cell monolayers exposed for 2, 6, 24, and 48 h to the materials. Data are expressed as means ± SEM (*n* = 3 independent preparations). *p < 0.05, ***p < 0.001, unpaired Student’s *t*-test. Cell uptake of GO (D) and FLG (E) (10 μg/mL) after 2 h exposure in the absence or presence of different endocytosis inhibitors (CPZ, CYTD, NOC). Means ± SEM, *n* = 3. ** *p* < 0.01, ***p < 0.001, one-way ANOVA/Tukey’s tests. All uptake measurements were done by SSC in flow cytometry and normalized over untreated cells (CTRL).

First, we evaluated the cellular uptake of GO and FLG. Figure 2A depicts representative transmission electron microscopy (TEM) micrographs showing GO and FLG internalized in the cell cytoplasm. The materials appear to be contained in intracellular vesicles (possibly early endosomes and lysosomes), consistent with the hypothesis of active endocytosis.^51^ Additional representative TEM images are reported in Figure S9.

The presence of such vesicles containing the two GRMs makes it possible to measure the uptake in a label-free fashion by measuring the side scattering (SSC) values by flow cytometry. These values are a recognized proxy for the increased cellular granularity upon material internalization.^52−55^ The instability of the RGO suspension in cell culture media (Figure S3) probably affected the material uptake. Due to the absence of uptake after 4 h of incubation (see Figure S10), RGO was excluded from the following experiments.

For both FLG and GO, the uptake kinetics were evaluated from 2 to 48 h, and the measured uptake was already significant after 2 h. For both materials, a plateau was reached after 24 h of incubation with 10 μg/mL of material, as shown in Figure 2C. FLG showed significantly higher SSC values compared to GO under the conditions applied. However, a quantitative comparison of the two materials might be challenging due to their different physicochemical (see absorption spectra in Figure S11) and mechanical properties. Indeed, SSC is mainly influenced by the augmented size of intracellular vesicles engulfing materials (augmented granularity of the cells), but the influence of the intrinsic scattering of the internalized material is unclear.

From a qualitative point of view, by TEM observation, the FLG-treated sample presented a remarkable number of holes in the cells, corresponding to some visible material accumulation. This might indicate that larger agglomerations of FLG are removed during the microtome slicing process (slice thickness is set as 70 nm). Such observations were less frequent in the case of GO-treated samples, possibly due to the lower dispersibility of FLG over time (see Figure S3) and to the different mechanical properties of the aggregates.

For a sufficiently dispersed subpopulation of smaller flakes, the endolysosomal uptake pathway might be involved in the internalization process, as for most nanomaterials. Therefore, we evaluated the GRM uptake in the presence of inhibitors of phagocytosis (actin depolymerization by cytochalasin D, CYTD), micropinocytosis (microtubule disruption by nocodazole, NOC), and clathrin-mediated endocytosis (chlorpromazine, CPZ).^56,57^ The working concentrations for the different inhibitors were selected according to the cell viability assay reported in Figure S12. For the selected concentrations, inhibition conditions were assessed using positive control uptake, as measured by flow cytometry and confocal imaging (see Figure S12B,C). Cells were pretreated for 30 min with the inhibitor and exposed to 10 μg/mL FLG and GO for 2 h before the evaluation of the uptake by flow cytometry. The results for both GO and FLG internalization pathways are reported in Figure 2D. For both materials, only the treatment with nocodazole significantly affected the uptake, indicating that micropinocytosis is the most likely internalization mechanism. Although the inhibitor platform poses some limitations due to the possible crosstalk between different pathways, this conclusion is in line with previous reports using GRMs of comparable size in other cell lines.^58−60^

## Translocation of GO and FLG across the 2D BBB Model

**Figure 3:**
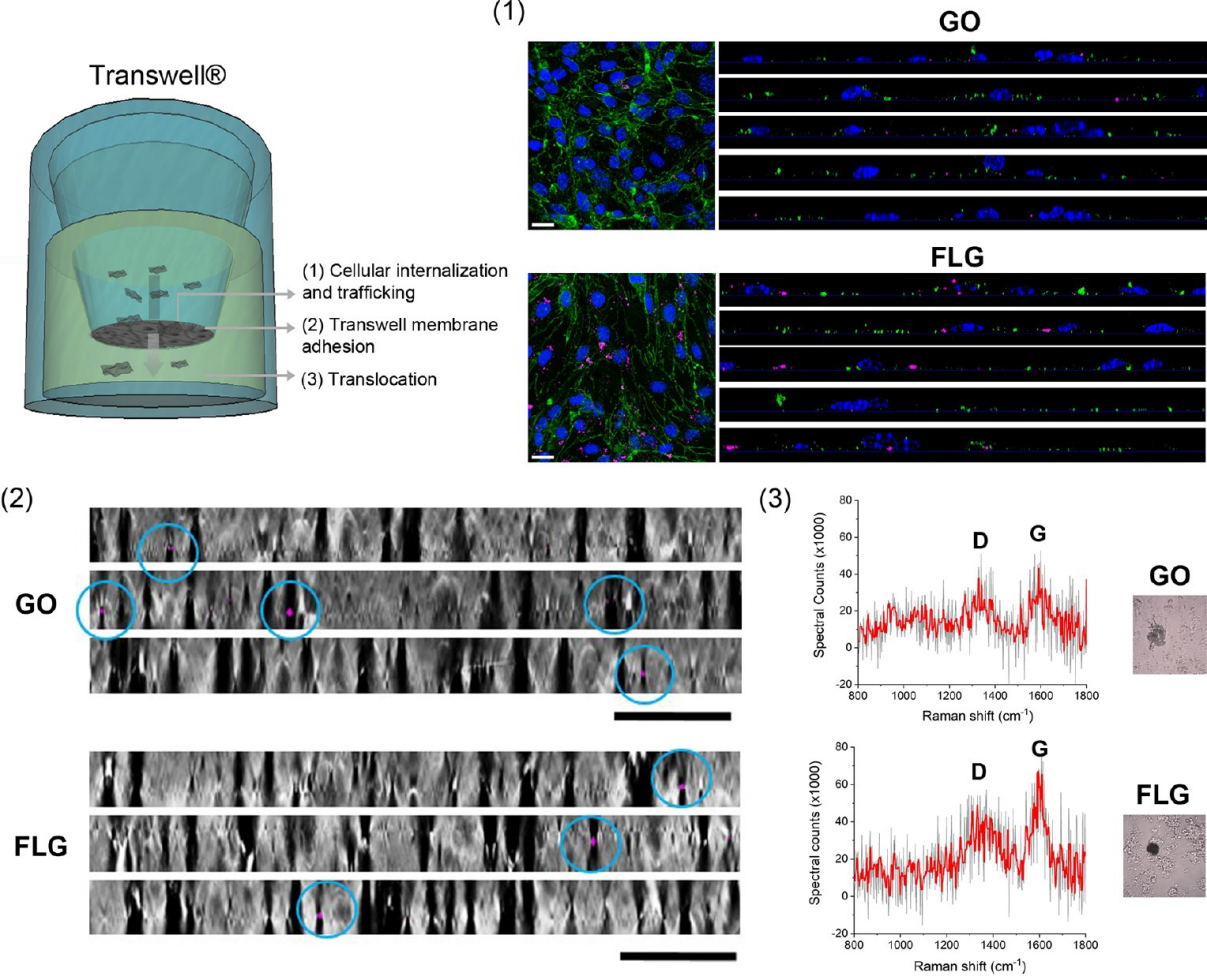
GO and FLG translocation across the bEnd.3 cell monolayer. (A) Schematic illustration of the journey of the two GRM flakes across the 2D BBB model. (B) Representative confocal *XY* planes and *Z* projections of bEnd.3 cells incubated with 10 μg/mL of GO or FLG for 24 h. Internalized graphene flakes are visible in pink (LR mode). Cells are stained with Hoechst (nuclei, blue) and ZO-1 antibodies (green). Scale bars: 50 μm. (C) Representative *Z* projections of Transwell membranes (BF) after cell removal. Some GO and FLG flakes translocating across the membrane are visible in pink (LR mode). Scale bars: 20 μm. (D) Analysis of the basolateral fractions: representative Raman spectra and corresponding sample area (BF images in insets) of the graphene agglomerates found in the basolateral fractions.

The translocation of fluorescent nanomaterials across the BBB through a transcellular way has been observed.^21,22,61^ Although the quantitative study of the translocation of label-free GRMs is a challenging task, some qualitative observations can be attempted. As schematically shown in Figure 3A, after 24 h of incubation with 10 μg/mL on the apical side, GO and FLG should first be internalized (endocytosed) in the cell layer, then extruded (exocytosed) and finally land into the basolateral fraction on the “brain” side of the Transwell barrier, characterized by a porous membrane of 3 μm pore size to allow culturing of the cells, while avoiding interferences with the passage of the two GRMs. In these experiments, it was essential to primarily assess the expression of tight junctions by the endothelial cells and measure high TEER values of the layer, to exclude the presence of holes and inhomogeneities in the barrier.

GO and FLG can be visualized by acquiring three-dimensional *Z*-stack images with the light reflection (LR) acquisition mode at the confocal microscope and positioning in the nuclear plane in order to detect flakes internalized in the intracellular space (Figure 3B), to detect the subpopulation of internalized flakes released by exocytosis and reaching the Transwell membrane. As shown in Figure 3C, we examined the translocation of GO and FLG across the Transwell membrane after cell removal. While some bigger graphene aggregates (and cell debris) were found on top, probably falling during the cell detachment process (Figure S13), we could spot some rare LR signals associated with the presence of graphene materials across the membrane.

Finally, we analyzed the basolateral fractions of the Transwell. The collected fractions were deposited on glass slides and observed at the optical microscope, where it was possible to spot a few dark agglomerates, as presented in representative insets in Figure 3D. We employed Raman spectroscopy to verify that the aggregates were actually the two GRMs, and two representative spectra for GO and FLG are reported. Despite the noise resulting from a background of organic material (medium, proteins, cell-derived vesicles, etc.), it was possible to appreciate the characteristic D (1350 cm^–1^) and G (1580 cm^–1^) bands with different relative intensities indicating the presence of GO and FLG.

## Graphene Uptake and Translocation in a 3D Human BBB Model: Multicellular Assembloids

We implemented a second, more realistic, human BBB 3D model based on human multicellular assembloids (hMCAs). hMCAs were prepared from 2D cultures of primary human astrocytes (NHA), human pericytes (hBVP), and human brain endothelial cells (hCMEC/D3) and validated as previously reported (and detailed in the Method section in the Supporting Information).^11,62^

**Figure 4:**
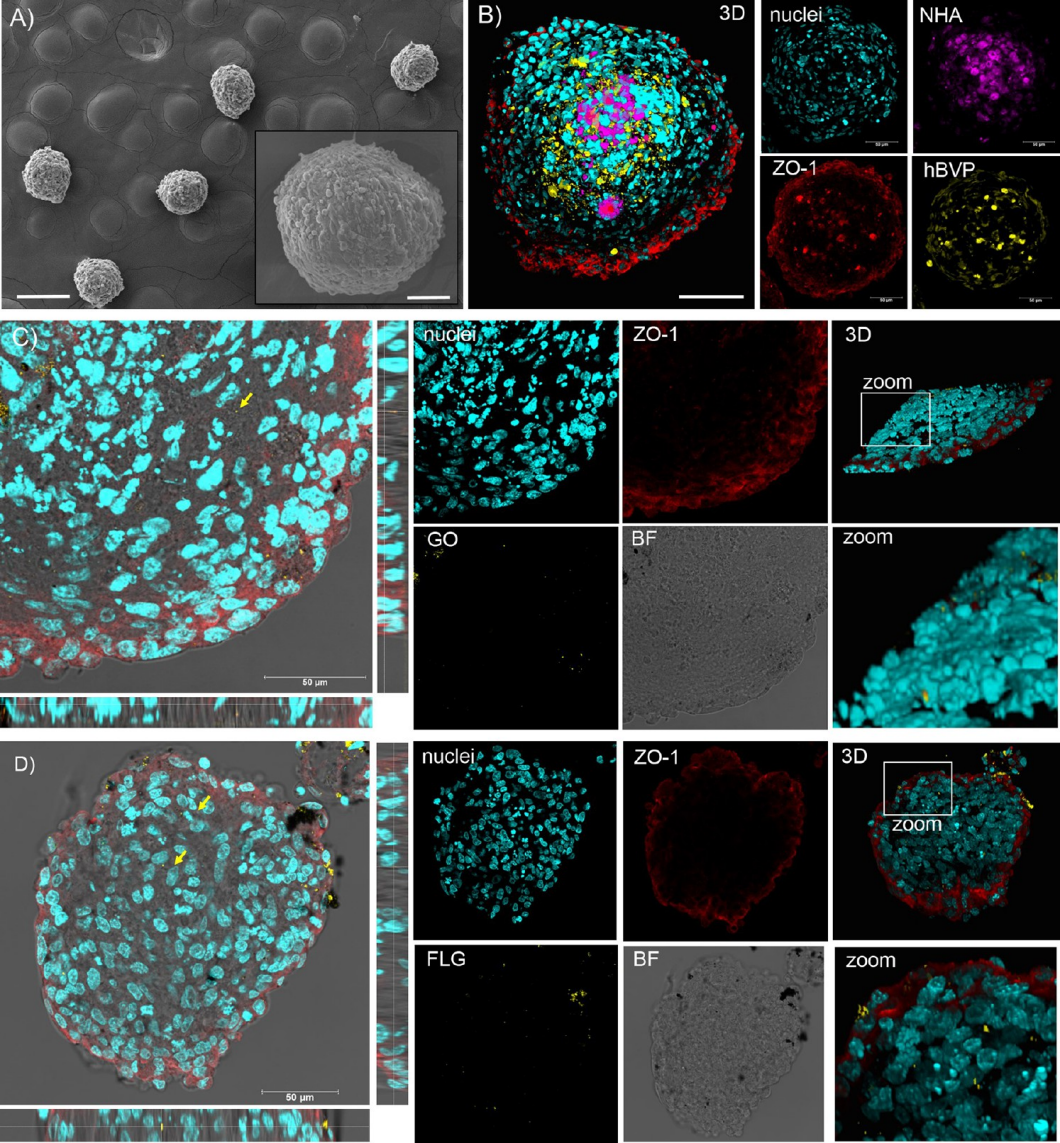
GO and FLG interactions with a 3D human multicellular assembloid model of BBB: SEM and confocal microscopy analysis. (A) SEM micrographs of hMCA showing their spherical morphology. (B) Confocal imaging and 3D reconstruction of hMCA: prestained NHA and hBVP are shown in purple and yellow, respectively; ZO-1 stained hCMEC/D3 tight junctions are shown in red. Representative confocal *XY* planes, *Z* projections, and 3D reconstructions from a 20 μm hMCA slice incubated with 10 μg/mL of GO (C) or FLG (D) for 24 h. Nuclei (Hoechst staining) are visualized in cyan, the two GRMs observed through LR mode are reported in yellow, and ZO-1 immunoreactivity is shown in red.

After 48–72 h of growth, the hMCAs appeared as spheroids of about 200 μm in diameter, as illustrated by the scanning electron microscopy (SEM) images of Figure 4A. In Figure 4B, the precise assembly architecture, as revealed by confocal microscopy, is shown. NHA and hBVP 2D cultures were stained using cell trackers before the hMCA formation, while hCMEC/D3 were stained after cryo-sectioning the whole hMCA, using ZO-1 immunofluorescence. The staining showed the specific organization of the assembloids, which are composed of an astrocytic core surrounded by a pericyte layer wrapped by endothelial cells sealing the periphery of the spheroid with tight junctions.

The assembloids were incubated with 10 μg/mL of either GO or FLG for 24 h. At the end of the incubation, about 50–90 organoids per condition were collected, washed to remove the excess external GO and FLG, fixed, and sliced at the cryostat. The slicing procedure allowed accessing the hMCA core for further confocal analysis. Figure 4C,D shows that large graphene aggregates adsorbed on the external layer of hMCA can deposit onto the slices during the slicing process, making the analysis in LR mode less reliable. Nevertheless, while moving across confocal planes, most graphene signals were found at the periphery of the spheroids, and only sporadic signals were spotted in the core, indicating the poor capability of BBB cells to exchange and pass over material exocytosed from the endothelial cell layer.

**Figure 5:**
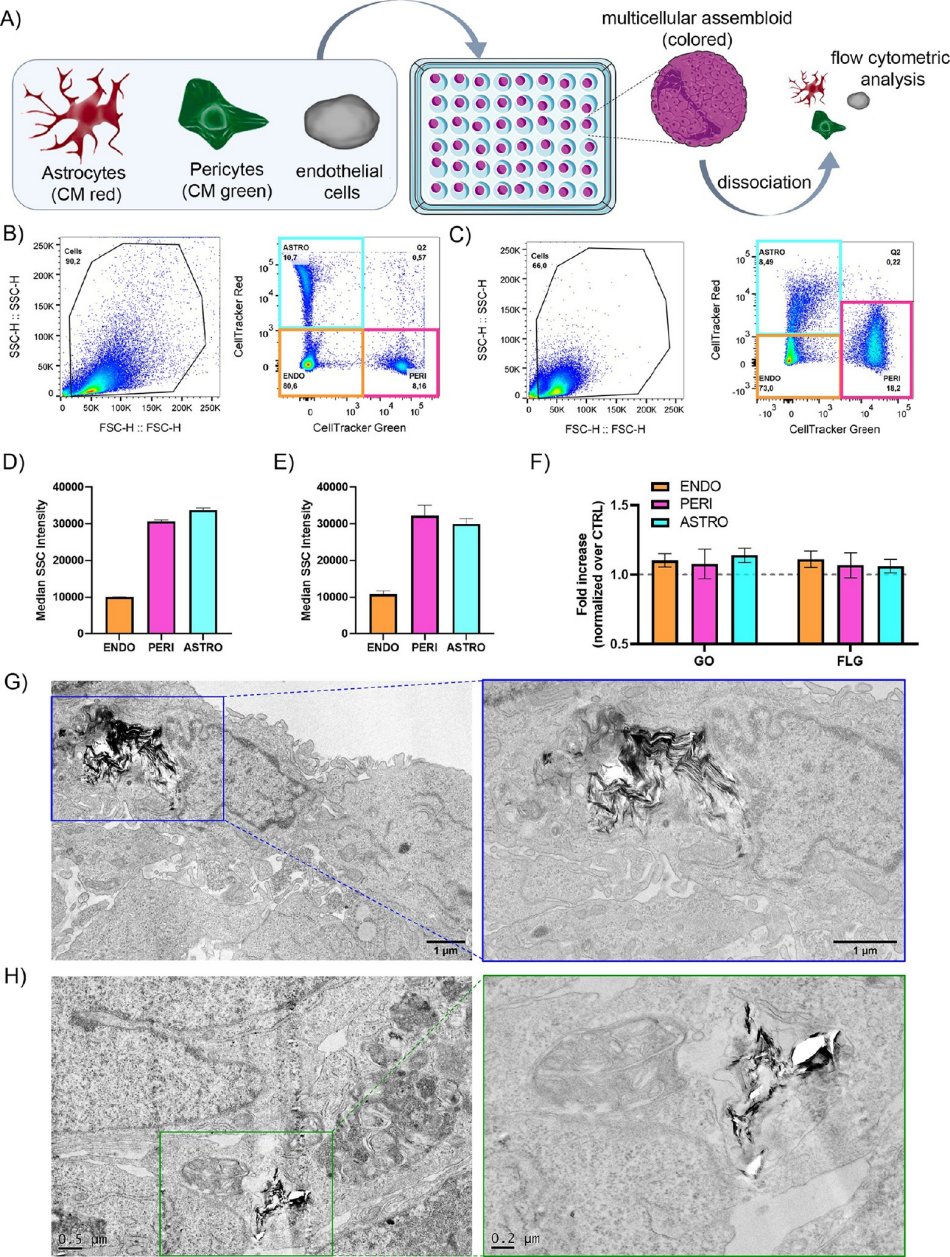
GO and FLG interactions with a 3D human multicellular assembloid model of BBB: flow cytometry and TEM analysis. (A) Schematic representation of the workflow for prestained hMCA formation and dissociation. (B) Dot plot of SSC vs FSC (forward scattering) and gating for cell tracker red (astrocytes) and green (pericytes) for the three independent cultures of NHA, hBPV, and hCMEC/D3 run together at the flow cytometer. (C) Dot plot of SSC vs FSC and gating for cell tracker red (astrocytes) and green (pericytes) for dissociated hMCA. (D) Mean SSC quantification for the three cellular cytotypes (NHA, hBPV, and hCMEC/D3) run independently at the flow cytometry. (E) Mean SSC quantification for the three cellular cytotypes (NHA, hBPV, and hCMEC/D3) after hMCA dissociation. Data are expressed as means ± SEM (*n* = 2). (F) Uptake of GO and FLG in the distinct cell cytotypes expressed as SSC fold increase over control cells. Data are expressed as means ± SEM (*n* = 3). TEM micrographs of hMCA exposed to GO (G) and FLG (H), showing rare material internalization in the peripheral cell layer.

We decided to complement our observations by employing flow cytometry and TEM imaging, as done in the 2D murine model. To evaluate the uptake of materials in the different layers of hMCA by SSC by flow cytometry, we dissociated the hMCAs after incubation with either GO or FLG and washing steps, as illustrated in Figure 5A. The protocol, detailed in Methods in the Supporting Information, was developed in house and validated using prestained NHA and hBVP that allowed separating clusters for the three cell populations, as shown in Figure 5B,C. The SSC basal values for control endothelial cells were significantly lower than those for astrocytes and pericytes in both single 2D cultures and dissociated hMCAs (Figure 5D,E), indicating a lower overall cell size. Furthermore, these data, together with the scatter plots in Figure 5C, showed that the cell morphologies were only slightly affected by the dissociation procedure. Measuring the SSC as fold increases over control cells for hMCA exposed to the two GRMs (Figure 5F) confirmed that no significant uptake and translocation of these materials occurred in a realistic 3D model of BBB.

TEM analysis further confirmed these observations. Although the micrographs depicted in Figure 5G,H show the occasional presence of flakes inside the cells, these images were very rare and required a long time of sample exploration (some other examples in Figure S14). These agglomerations are localized in cells situated at the periphery of the slice that, according to the architectural organization of hMCA, correspond to the endothelial cell layer. No sign of GO and FLG in the internal core was found by TEM analysis. Even in this case, it can be noticed how FLG (and not GO) produced bigger/stiffer agglomerates, producing holes when encountering the blade during the microtome slicing procedure.

The interaction of two different colloidal GRMs, GO and FLG, with BBB in *in vitro* models of increasing complexity has been thoroughly investigated. GO and FLG do not induce any obvious harm to the BBB cells in terms of viability, tight junction expression, barrier morphology, and functionality, adding an essential brick to the safe use of graphene in neuroscience. However, despite their excellent cargo capability and ease of functionalization, our GRMs do not seem to be promising carriers for BBB translocation.

In this work, we employed an array of complementary techniques, all label-free, to study GO and FLG behavior in 2D and 3D models of BBB. By means of confocal microscopy, flow cytometry, and electron microscopy, we found that graphene is internalized by endothelial cells, depending on its dispersion state, in large vesicles, which is compatible with the micropinocytosis uptake mechanism. However, the export and the cell-to-cell exchange of this material rarely occur, especially in more sophisticated models of primary human cells mimicking the complexity of the 3D BBB architecture.

The workflow developed here can be applied to any fluorescent or label-free nanomaterial and will ensure thorough screening of potential nanocarriers to the brain, bypassing costs and ethical concerns of *in vivo* investigations. This work, complementing the existing *in vivo* literature, can contribute to guiding the graphene community to focus their efforts toward graphene use more oriented to regenerative medicine, prosthetics, and sensors, rather than pure nanomedicine.^63−66^

## Supporting Information Available

Materials and methods and additional figures on materials characterization, *in vitro* experiments, imaging and mass spectrometry analysis. The Supporting Information is available free of charge at https://pubs.acs.org/doi/10.1021/acs.nanolett.3c00377.

Materials and methods and additional figures on materials characterization, *in vitro* experiments, imaging and mass spectrometry analysis (PDF)
Supplementary File P1: Full list of quantified proteins (XLSX)
Supplementary File P2: Full sets of altered proteins (XLSX)
Supplementary File P3: Full sets of altered proteins (XLSX)
