# Supplementary material for: Interactions of Graphene Oxide and Few-Layer Graphene with the Blood–Brain Barrier
Source: Nano Lett. 2023 Mar 14;23(7):2981–90. doi: 10.1021/acs.nanolett.3c00377 (PMC10103300; doi:10.1021/acs.nanolett.3c00377)
Supplement: Supplementary file 1 — nl3c00377_si_001.pdf [file nl3c00377_si_001.pdf]

Supporting Information for:

Interactions of graphene oxide and few-layer graphene with the blood-brain barrier

Valentina Castagnola[§1,2*], Lieselot Deleye[§1], Alice Podestà[1], Edra Jaho[1], Fabrizio Loiacono[2], Doriana Debellis,[3] Martina Trevisani[1], Dinu Zinovie Ciobanu[4], Andrea Armirotti[4], Francesco Pisani[1,5], Emmanuel Flahaut[6], Ester Vazquez[7,8], Mattia Bramini[1,9], Fabrizia Cesca[1,10], and Fabio Benfenati[1,2*]

1. Center for Synaptic Neuroscience and Technology, Istituto Italiano di Tecnologia, Largo Rosanna Benzi 10, 16132, Genova, Italy
2. IRCCS Ospedale Policlinico San Martino, Largo Rosanna Benzi 10, 16132, Genova, Italy
3. Electron Microscopy Facility, Istituto Italiano di Tecnologia, Via Morego, 30−16163, Genoa, Italy
4. Analytical Chemistry Lab, Istituto Italiano di Tecnologia, Via Morego 30, 16163, Genova, Italy
5. Department of Biosciences, Biotechnologies and Biopharmaceutics, University of Bari "Aldo Moro", Bari, Italy
6. CIRIMAT, UMR 5085, CNRS-INP-UPS, Université Toulouse 3 Paul Sabatier, 118 route de Narbonne, F-31062 Toulouse cedex 9, France
7. Instituto Regional de Investigación Científica Aplicada (IRICA), Universidad de Castilla-La Mancha, 13071, Ciudad Real, Spain
8. Facultad de Ciencias y Tecnologías Químicas, Universidad de Castilla-La Mancha, Avda. Camilo José Cela S/N, 13071, Ciudad Real, Spain
9. Department of Cell Biology, Universidad de Granada, C. Fuentenueva s/n, 18071, Granada, Spain
10. Department of Life Sciences, University of Trieste, 34127, Trieste, Italy

Supplementary Methods

GRM materials. FLG was prepared as described in our previous works.[1-2] GO was supplied from Antolin group (Burgos) and washed several times with water to obtain a final pH = 5. RGO was prepared from the reduction of GO (Antoling group, Burgos) in H_2 atmosphere with a hydrogen flow rate of 5 $L{\cdot}h^{-1}$ at 800° C, as described elsewhere.[3]

Turbiscan Analysis. The stability of the suspensions was evaluated using Turbiscan LAB (Formulation) equipment at room temperature. Suspensions were prepared at 10 mg/L in cell culture media (complete DMEM, total volume 20 mL) and placed in glass vials before performing analysis. Both transmission and reflection were monitored *vs.* time for 48 h (1 scan per minute).

Cell culture. Immortalized brain endothelial cells (bEnd.3 [***BEND3***] (ATCC® CRL-2299™) were cultured in Dulbecco's modified eagle medium (DMEM) supplemented with 10% FBS, 1% Penicillin/Streptavidin, and 1% Glutamine. Cells were grown in T75 culture flasks in a humidified atmosphere of 5% CO_2 and 90% humidity at 37 °C. Cell culture medium was replaced every two days, and cells were maintained between passages 25 and 30. For the monolayer, cells were seeded onto 150 µg/mL collagen-coated upper Transwell® membranes (12mm Ø inserts, pore size 3.0 µm, growth area 1.12 cm^2) or onto 18 mm Ø glass coverslips at 40,000 cells per well. Cell culture medium was replaced twice a week. All reagents were purchased from Thermo Fisher unless specified differently.

Cell exposure to FLG, GO, and RGO. FLG, GO, and RGO water dispersions were placed in an ultrasonic bath (Labsonics LBS2, Falc, Italia) for 10 min and then dispersed in complete cell medium at a concentration of 10 µg/mL. Cells were exposed to GRMs by replacing their medium with the FLG, GO, and RGO dispersions.

Cell viability assays. bEnd.3 monolayers were exposed to 10 µg/mL of the three GRMs for 24 h. Cells were stained with propidium iodide (PI, 1 µM) for cell death quantification, calcein-AM (2 µM) for cell viability, and Hoechst 33342 Fluorescent Stain (1 µM) for nuclei visualization for 3 min at room temperature (RT). Cell viability was quantified by imaging cultures at 20x (0.5 NA) magnification using a Nikon Eclipse-80i upright epifluorescence microscope with a random sampling of 10 fields per image. Image analysis was performed using the ImageJ software.

Uptake inhibitors platform setting. MTT viability assay was performed to establish the appropriate concentration of the inhibitors. Briefly, cells were seeded at 10^4 cells/well in a 96-well plate (Corning, #CLS3997, Sigma-Aldrich, USA). Cells were grown for 24 h and then incubated with chlorpromazine hydrochloride (#C8138, Sigma-Aldrich, USA), cytochalasin D (#C2618, Sigma-Aldrich, USA), and

nocodazole (#M1404, Sigma-Aldrich, USA) at different concentrations (ranging from 3 to 400 μM) for 2 hours. After incubation, the medium was removed and replaced with 50 μL Serum-Free medium and 50 μL MTT Reagent for each well. Cells were incubated at 37 °C for 3 h. Then, the medium was removed and replaced with 150 μL of MTT solvent for each well. Cells were incubated for 15 min in the dark with gentle shaking. Absorbance values at 590 nm were obtained using a Tecan Infinite F500 (Tecan, Switzerland) multimode microplate reader.

Trans-endothelial electrical resistance measurements. Trans-endothelial electrical resistance (TEER) measurements were performed on bEnd.3 monolayers using a CellZscope+ instrument (NanoAnalytics, Münster, Germany).

4 kDa fluorescein isothiocyanate-dextran transport studies. Transport studies were performed by measuring the 4 kDa Fluorescein isothiocyanate–dextran (FD4, #46944, Sigma-Aldrich) permeability on cells at 37 °C at 20-120 min. Transport studies were performed after 6 and 48 h FLG, GO, and RGO flake exposure. Before each experiment, the Transwell® apical medium was replaced with 200 μg/mL FD4 in PBS and the basolateral compartment medium with PBS. At the indicated time points, 100 μL aliquots were sampled from the basolateral chamber into a 96 wells plate and immediately replaced by 100 μL of fresh PBS to maintain the total volume. Fluorescence measurements of the samples were conducted using a Tecan Infinite F500 (Tecan, Switzerland) multimode microplate reader using the 485 nm FITC channel for excitation and reading the fluorescence emission at 535 nm. Serial dilutions of FD4 in the range of 0-200 μg/ml in PBS were prepared to obtain a calibration curve. Linear regression was applied to define the correlation between fluorescence intensity and FD4 mass concentration and used to determine the total mass of FD4 in the basolateral chamber. The regression coefficients obtained from the linear curve fits were generally 0.98-0.99 (n=3 wells, from 3 independent culture preparations). The apparent permeability (P_{app}) of FD4 was determined using the equation:

$$P_{app} = \frac{1}{A} C_0 \frac{dQ}{dt}$$

where A represents the surface area of the membrane in cm^2, C_0 is the initial concentration of FD4 in the apical compartment (μg/mL), and dQ/dt is the amount of FD4 that appears in the basolateral compartment in the given time period (μg/min).

Immunofluorescence staining. bEnd.3 monolayers were fixed in 4% paraformaldehyde in PBS for 15 min at room temperature (RT). Cells were permeabilized with 0.1% Triton X-100 for 5 min, blocked with 2% bovine serum albumin (BSA)/0.05% Tween 80 for 30 min at RT, and incubated

with primary antibodies in the same buffer overnight. Primary antibodies used were: rabbit monoclonal anti-zona occludes 1:200 (ZO1, #61-7300, Termo Fisher Scientific), rabbit polyclonal anti-claudin 5 (CLD5, # PA599415, Thermo Fisher Scientific), and rabbit monoclonal anti-vascular endothelial cadherin (VE-cad, #36-1900, Thermo Fisher Scientific). After the primary antibody incubation and several PBS washes, cells were incubated for 45 min with the appropriate secondary antibodies purchased from Molecular Probes (Thermo-Fisher Scientific) and diluted 1:500 in the blocking buffer solution. Cells were then incubated for 5 min with Hoechst (1 μM, #3342, Sigma). Samples were mounted with Vectashield antifade mounting medium (#H-1000-10, Vector Laboratories) on microscope slides.

For hMCA slices deposited onto PLL/Gelatin coated microscope glass slides (see hMCA cryo-sectioning) a ImmEdge Hydrophobic Barrier PAP Pen was used to delimitate the slice area. The slices were washed 3X with PBS for 5 min then blocked and permeabilized for 1 h at RT with a solution of 10% w/v BSA and 0.3% v/v Triton X-100 in PBS. Rabbit monoclonal anti-zona occludens 1:200 (ZO1, #61-7300, Termo-Fisher Scientific) was used in a solution 2% w/v BSA and 0.1% v/v Triton X-100 in PBS and incubated overnight at 4° C. Samples were washed 3X with PBS for 5 min the incubated with fluorescently conjugated secondary antibodies of choice in the same blocking/permeabilizing solution for 2 h at RT. Samples were finally washed 3X with PBS, stained with Hoechst, and mounted with Vectashield antifade mounting medium as described above.

Confocal imaging. Image acquisitions were performed using a confocal microscope (Leica SP8 TCS, Leica Microsystems GmbH, Wetzlar, Germany) with 40X (1.3 NA) and 63X (1.4 NA) oil immersion objectives. Confocal images were acquired by using 1024x1024 scan format at 200 Hz speed. Offline analysis was performed using LasX and ImageJ software.

Sample preparation for proteomic analysis. bEnd.3 cells were plated at a density of 80,000 cells in completed DMEM medium (Dulbecco's Modified Eagle Medium supplemented with 10% fetal bovine serum, 1% penicillin-streptomycin, and 1% Glutamine) in a 12-wells tissue culture. Confluent cells were incubated with FLG and GO (10 μg/mL) for 24 h at 37 °C in a 5% CO_2 atmosphere. Previously, the FLG and GO clusters were dispersed using a bath sonicator (Labsonics LBS2, Falc) at a frequency of 59 Hz and 100% of power for 15 min. After graphene exposure, the medium was removed, and bEnd3 cells were washed three times in PBS to remove clusters of graphene attached to the cellular surface. Then, cells were treated with RIPA buffer (10 mM Tris-HCl pH 7.4, 140 mM NaCl, 1 mM EDTA, 0.5 mM EGTA, 1% Triton X-100, 0.1% sodium dodecyl sulfate (SDS), and 0.1% sodium deoxycholate) supplemented with complete EDTA-free protease inhibitors (Roche Diagnostics) and serine/threonine and tyrosine phosphatase inhibitors (Sigma). Lysates were

sonicated (Branson SLPe sonicator; 25% amplitude) and centrifuged for 15 min minutes at 4 °C at 13000 RPM. Supernatants were collected, and the total protein content was determined with the BCA Protein Assay kit (Thermo Scientific) using the standard protocol. The samples were stored at -20 °C.

Following BCA protein quantification, aliquots of 50 µg of total protein were collected from each sample. Cysteine residues were reduced in 100 mM dithiothreitol (10 µL, 56 °C for 30 min) and then alkylated with 100 mM iodoacetamide (30 µL) at RT in the dark. Proteins were then digested with trypsin (1:50 w/w ratio with the sample) overnight at 37 °C under gentle shaking. The resulting peptides were then dried under vacuum and resuspended in 100 µL of 3% acetonitrile solution at the time of proteomic analysis.

Mass Spectrometry data acquisition and analysis. The proteomics analysis was conducted on a Thermo Exploris 480 orbitrap system coupled with a Dionex Ultimate 3000 nano-LC system. After trapping and desalting, the tryptic peptides were loaded on a Aurora C18 column (75 mm x 250 mm, 1,6 µm particle size) nanocolumn (Ion Opticks, Fitzroy, Australia) and separated using a linear gradient of acetonitrile in water (both added with 0.1% formic acid), from 3% to 41% in 1 h, followed by column cleaning and reconditioning. The flow rate was set to 300 nL/min. Total run time was 1.5 h. Injection volume was set to 1 µL. Peptides were analyzed in positive ESI mode, using a capillary voltage set to 2.0 kV. The RF lens was set to 40% and the AGC target was set to 300%. Data acquisition was performed in Data Independent mode (DIA) with a survey scan set from 400 to 1000 m/z at 120,000 resolution, followed by MS/MS acquisition of 60 m/z transmission windows, each having a fixed 10 Da width. MS/MS spectra were acquired in HCD mode.
All the collected MS/MS spectra were analyzed using Spectronaut,[4] by running a DirectDIA analysis against the reference *Mus Musculus* FASTA database (Tax ID: 10090 reporting 17126 reviewed entries). Cysteine carbamidomethylation was selected as fixed modification; acetylation, methylation, deamidation, oxidation and phosphorylation were selected as variable modifications. Positive protein identifications were retained at 1% false discovery rate (FDR) threshold and at least two peptides were used for protein quantification.

Uptake studies by flow cytometry. GRM uptake was evaluated using side scatter (SSC) values as measured by flow cytometry MaxQuant Analyzer 10 (Miltenyi) flow cytometer. Data were analyzed with Flow Jo 10.8. bEnd.3 cells exposed to the three GRMs (and control cells) were washed multiple times with PBS to remove aggregates deposited onto the cells' surface and detached using trypsin. Cells from a 12-well plate were centrifuged, resuspended in 200 µL of PBS in flow cytometry tubes, and run without further fixation. A minimum of 15,000 cells per samples were analyzed.

For hMCA dissociation, NHA were pre-stained with CellTracker™ Red CMTPX Dye (Invitrogen, #C34552), and hBVP were pre-stained with CellTracker™ Green CMFDA Dye (Invitrogen, #C2925). Flow cytometry analysis was performed using a 635 nm excitation laser and collecting emission with a bandpass filter 655-730 nm to detect NHA cells. A 488 nm excitation laser was used to isolate hBVP signal and the emission was collected using a bandpass filter 525/50 nm.
For all the analyses, debris, dead cells, and large graphene aggregates were excluded by gating over physical parameters (FSC/SSC).

Raman Spectroscopy. Raman spectra were measured by a Renishaw inVia Raman spectrometer with a Nikon 100x water immersion objective and a 1.0 NA delivering a 532 nm laser with 10% power. To obtain a representative mean, each spectrum was collected for 1 s and accumulated five times.

Absorption Spectroscopy. Absorption spectra of GO and FLG were acquired using a Cary 5000 UV-Vis-NIR Spectrophotometer (Agilent).

Human Multicellular Assembloid (hMCA) preparation. Each assembloid was grown in a single well from a 96-well plate. First, the wells were coated with 1% (wt/vol) agarose LE at a volume of 50 µl. Then, for confocal imaging and flow cytometry applications, human brain vascular pericytes (hBVPs, ScienCell) and human astrocytes (NHA, Lonza) were labeled with CellTracker Green CMFDA Dye and CellTracker Red CMTPX Dye, respectively (Thermo Fisher Scientific). For TEM analysis, cells were not labeled. The EGM-2 Endothelial medium bulletkit (Lonza), was used for seeding endothelial cells (hCMEC/D3, Cedarlane) and also as hMCA medium. After these pretreatments, the three cell lines, hCMEC/D3, hBVPs, and NHA, were detached and counted as previously described.[5-6] Subsequently, the three cell lines were seeded at a 1:1:1 ratio, at a density of 1.5 x 10^3 cells per well (for each cytotype) in a final volume of 200 µl BBB working medium. For the next 48 h they were allowed to self-assemble under standard cell growth conditions (37 °C, 5% CO_2, 95% humidity).

Scanning Electron Microscopy. hMCA were established for 24-72 h and treated with either GO or FLG for 24 h. hMCA were then collected and pooled in Netwell inserts with 74 µm polyester mesh bottoms attached to polystyrene rings (Corning, Product Number 3477). The samples were rinsed three times in PBS then fixed with 2 % v/v glutaraldehyde in 0.1 M sodium cacodylate buffer for 2 h at RT. hMCA were then washed three times in 0.1 M cacodylate buffer for 10 min, followed by two washes in MilliQ water for 5 min. Following the cell fixation, the samples were subjected to sequential dehydration in graded ethanol (30, 50, 70, 90, 96, and 100 % v/v). Each step was repeated twice. After ethanol dehydration, the samples were rinsed in a solution of hexamethyldisilazane (HMDS) and ethanol at increasing concentrations (25, 50, and 75 % v/v) for 10 min each. Finally,

samples were immersed in pure HMDS for 30 min and left under the fumehood overnight to let the HMDS evaporate completely. Unless otherwise indicated, all chemicals were purchased from Sigma Aldrich.

Transmission Electron Microscopy. TEM imaging was performed on bEnd.3 monolayer cell cultures and on hMCA. In both cases, after incubation with the GRMs, cells (or spheroids) were washed 3 times with PBS, detached (only in the case of bEnd.3 cells) and pelleted by gentle centrifugation on the bottom of a 1.5 mL Eppendorf tube. Then, cells were fixed in 0.1 M sodium cacodylate buffer at pH 7.4 containing 1.5% glutaraldehyde for 1 h. For hMCA a mixture of 1.5% glutaraldehyde and 1.5% paraformaldehyde was used to enhance the penetration. After this procedure, the cells were post fixed in the buffer described above containing 1% osmium tetroxide and stained with an aqueous solution containing 1% uranyl acetate. After progressive dehydratation in increasing %v/v of ethanol, cells were embedded in epoxy resin (Epon 812, TAAB) and sliced using a diamond blade (Diatom) with an ultramicrotome (UC6, Leica). TEM micrographs were obtained using a JEOL JEM 1011, 100 kV acceleration voltage.

hMCA cryosectioning. At least 48 hMCAs per condition were collected in a 1.5 ml Eppendorf and washed 3X in PBS by mild centrifugation. Samples were then fixed 4% paraformaldehyde in PBS for 15 min at RT. After fixing, the hMCA were washed with 3X with fresh PBS. The PBS was then removed and replaced with O.C.T compound (Scigen scientific Inc.). Next, the Eppendorf bottom was placed on dry ice. Immediately, the back of a cotton swab stick was immersed halfway into the O.C.T and held straight until the O.C.T. became solid. Next, the solid O.C.T. that had encapsulated the assembloid pellet, was pulled out of the Eppendorf using the back of the stick. This created a "popsicle" with the hMCA pellet located at the tip. Then, the stick was cut off, leaving only a short part protruding out from the O.C.T., and the sample was glued onto a cryostat sample holder using more O.C.T. Finally, 20 µm-thick slices were cut from the assembloid pellet and adhered onto PLL/Gelatin-coated microscope glass slides and stored at -20 °C until further processing (see immunofluorescence staining).

hMCA dissociation. About 30 hMCAs per condition were pooled in a 1.5 mL Eppendorf, washed 3X in PBS, then incubated at 37 °C under constant gentle shaking for 90 min in 20 U of Papain (from papaya latex, # P4762, Sigma-Aldrich) diluted in preheated Hank's balanced salt solution (HBSS, #14025092, Thermo Fisher Scientific). After incubation, the residual enzyme was washed off with PBS, and 1 mL of PBS was left for the subsequent dissociation step. hMCAs were mechanically dissociated back into individual cells using a 22G 3.8-cm, 40mm long needle (BD Microlance) and a 5 mL syringe. Next, the cells were pelleted by centrifugation at 200 x g for 5 min, and 800 µL of the

supernatant was removed. The pellet was resuspended in the remaining PBS and transferred to a round bottom polystyrene tube (Falcon, Corning) for flow cytometry analysis.

References

1. Braccia, C.; Castagnola, V.; Vázquez, E.; González, V. J.; Loiacono, F.; Benfenati, F.; Armirotti, A., The lipid composition of few layers graphene and graphene oxide biomolecular corona. *Carbon* **2021,** *185*, 591-598.
2. Liessi, N.; Maragliano, L.; Castagnola, V.; Bramini, M.; Benfenati, F.; Armirotti, A., Isobaric labeling proteomics allows a high-throughput investigation of protein corona orientation. *Analytical chemistry* **2020,** *93* (2), 784-791.
3. Evariste, L.; Lagier, L.; Gonzalez, P.; Mottier, A.; Mouchet, F.; Cadarsi, S.; Lonchambon, P.; Daffe, G.; Chimowa, G.; Sarrieu, C., Thermal reduction of graphene oxide mitigates its in vivo genotoxicity toward xenopus laevis tadpoles. *Nanomaterials* **2019,** *9* (4), 584.
4. Bruderer, R.; Bernhardt, O. M.; Gandhi, T.; Miladinović, S. M.; Cheng, L.-Y.; Messner, S.; Ehrenberger, T.; Zanotelli, V.; Butscheid, Y.; Escher, C., Extending the limits of quantitative proteome profiling with data-independent acquisition and application to acetaminophen-treated three-dimensional liver microtissues*[S]. *Molecular & Cellular Proteomics* **2015,** *14* (5), 1400-1410.
5. Bergmann, S.; Lawler, S. E.; Qu, Y.; Fadzen, C. M.; Wolfe, J. M.; Regan, M. S.; Pentelute, B. L.; Agar, N. Y.; Cho, C.-F., Blood–brain-barrier organoids for investigating the permeability of CNS therapeutics. *Nature protocols* **2018,** *13* (12), 2827-2843.
6. Pisani, F.; Castagnola, V.; Simone, L.; Loiacono, F.; Svelto, M.; Benfenati, F., Role of pericytes in blood–brain barrier preservation during ischemia through tunneling nanotubes. *Cell death & disease* **2022,** *13* (7), 1-14.

Supplementary Figures

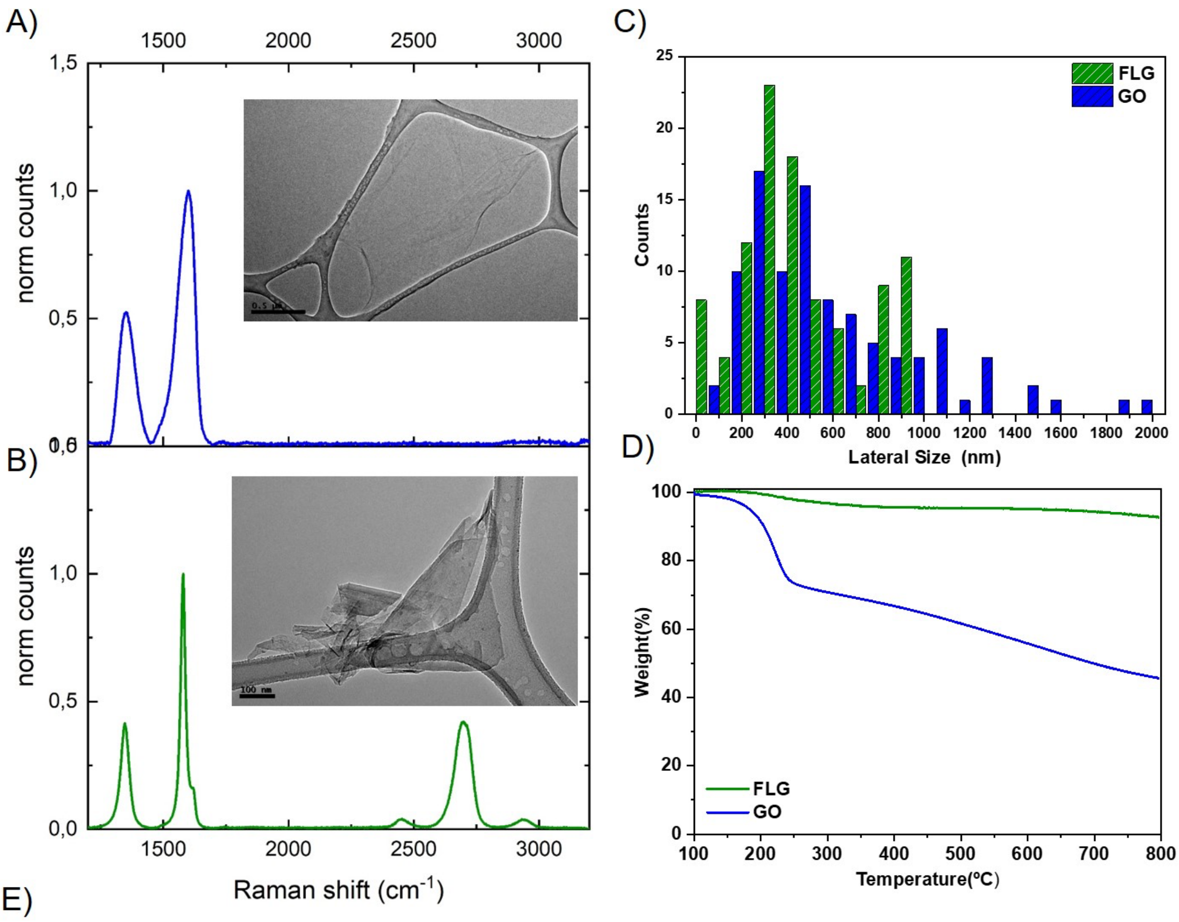

E)

Sample	%C	%H	%N	%S	%O	TGA weight lost (%)	(nm)
FLG	91.62 ±0.43	0.79 ±0.02	0.55 ±0.02	0.51 ±0.02	6.53 ±0.12	4.81	300±23
GO	51.12 ±0.72	2.92 ±0.03	0.12 ±0.01	0.05 ±0.007	45.79 ±0.19	44.00	672±39

Figure S1. Characterization of GO and FLG. Raman spectrum (at 532 nm) and representative HRTEM imaging (inset) of A) GO and B) FLG. C) Statistical size distribution form HRTEM images, D) TGA in nitrogen atmosphere and, E) elemental analysis of FLG and GO.

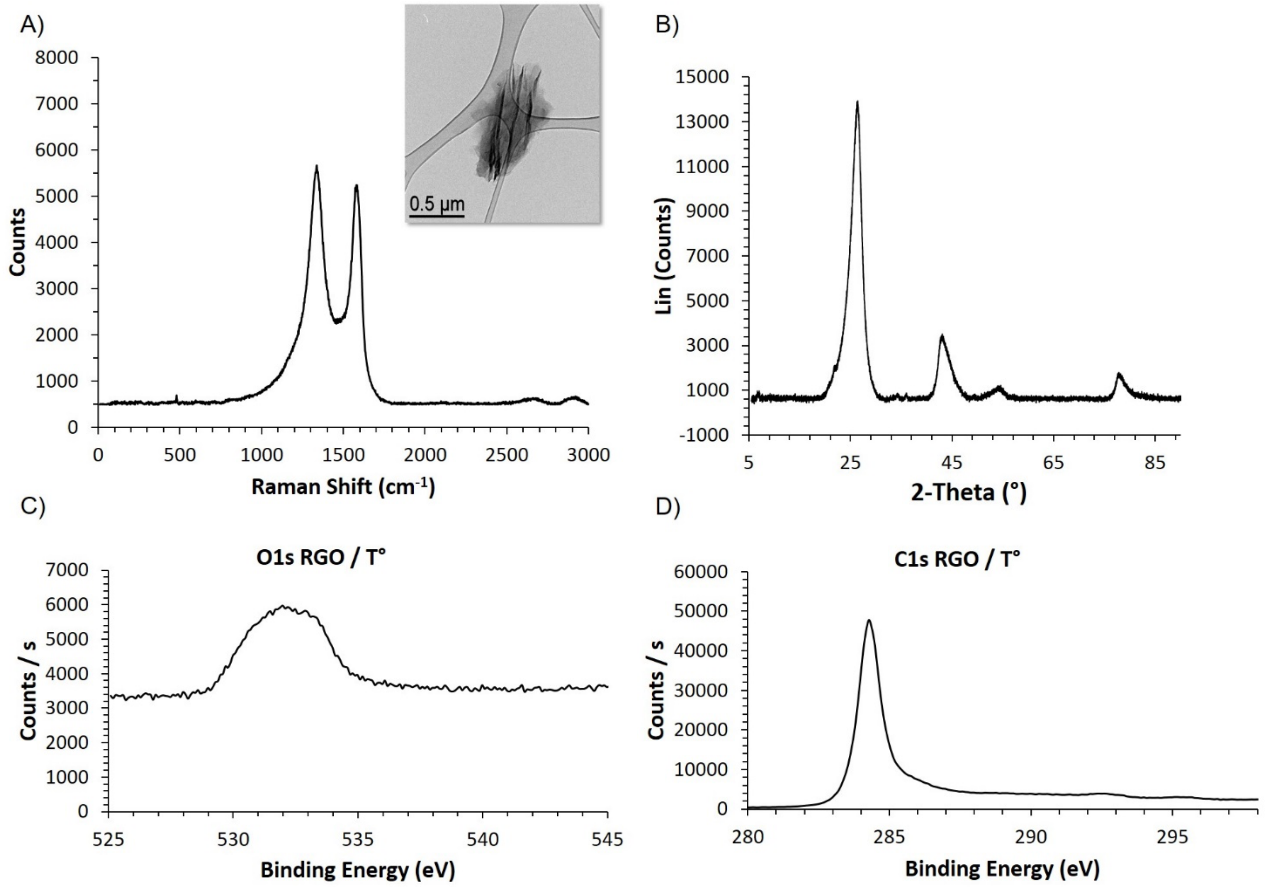

Figure S2. Characterization of RGO. A) Raman spectrum (at 532 nm) and representative HRTEM imaging (inset). B) XRD analysis. C) O1s XPS spectrum (5%) and D) C1s XPS spectrum (95%).

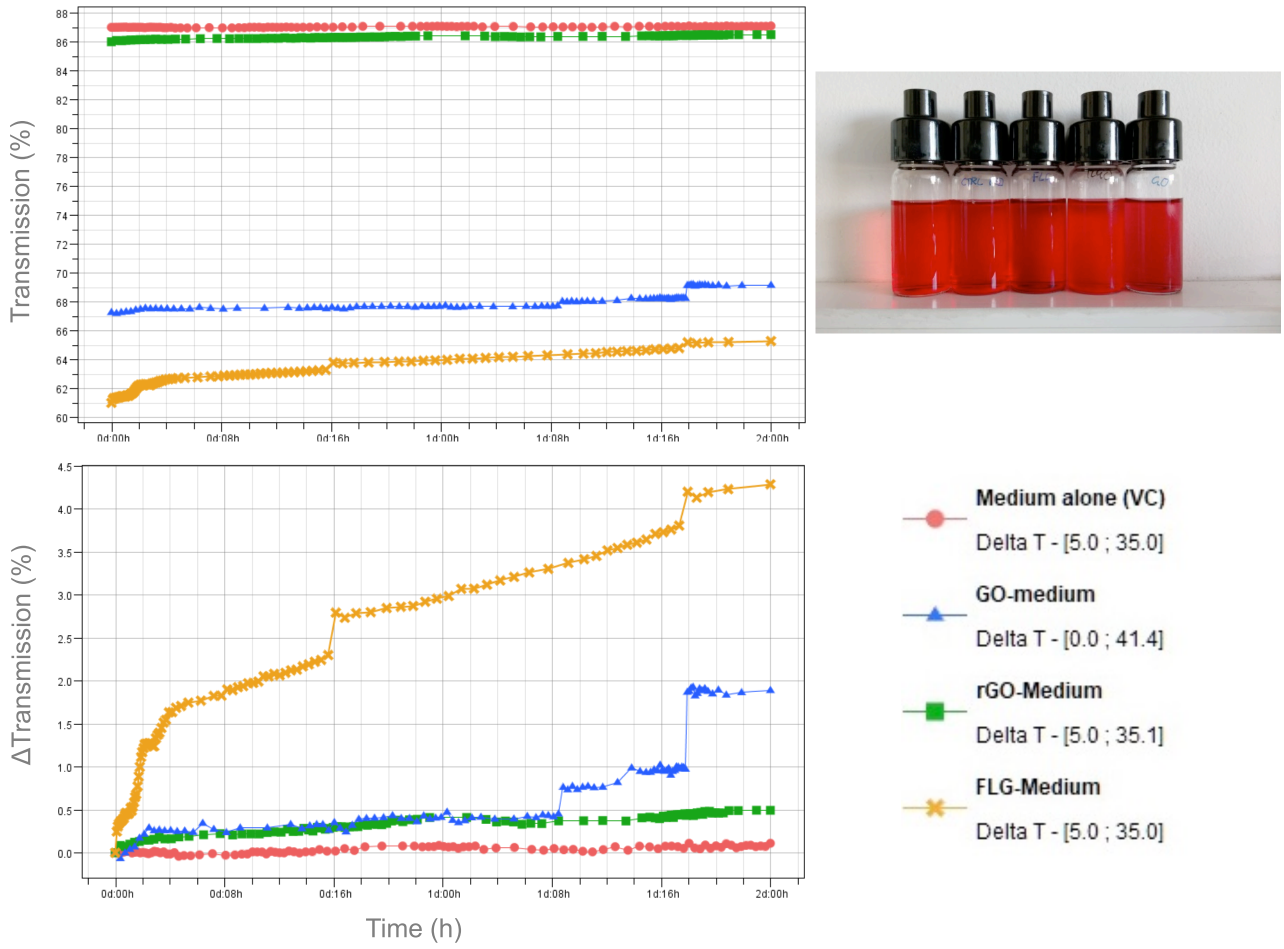

Figure S3. Stability analysis (Turbiscan) of the three GRMs in cell culture medium. *Upper panel:* Light transmission (T%) over time that is inversely proportional to the turbidity of the sample analyzed. Low transmission (FLG, GO) indicates the presence of a colloidal dispersion. High transmission indicates the absence of a colloidal dispersion (medium alone) or dispersions that are fully precipitated before the initial time point (RGO). *Lower panel:* ΔT (%) over time, indicating the tendency of FLG to deposit overtime (ΔT increase). ΔT remains stable for samples with high initial transmission (medium and RGO). On the *top right, a* picture of the dispersions in cell culture media (complete DMEM) prior to analysis.

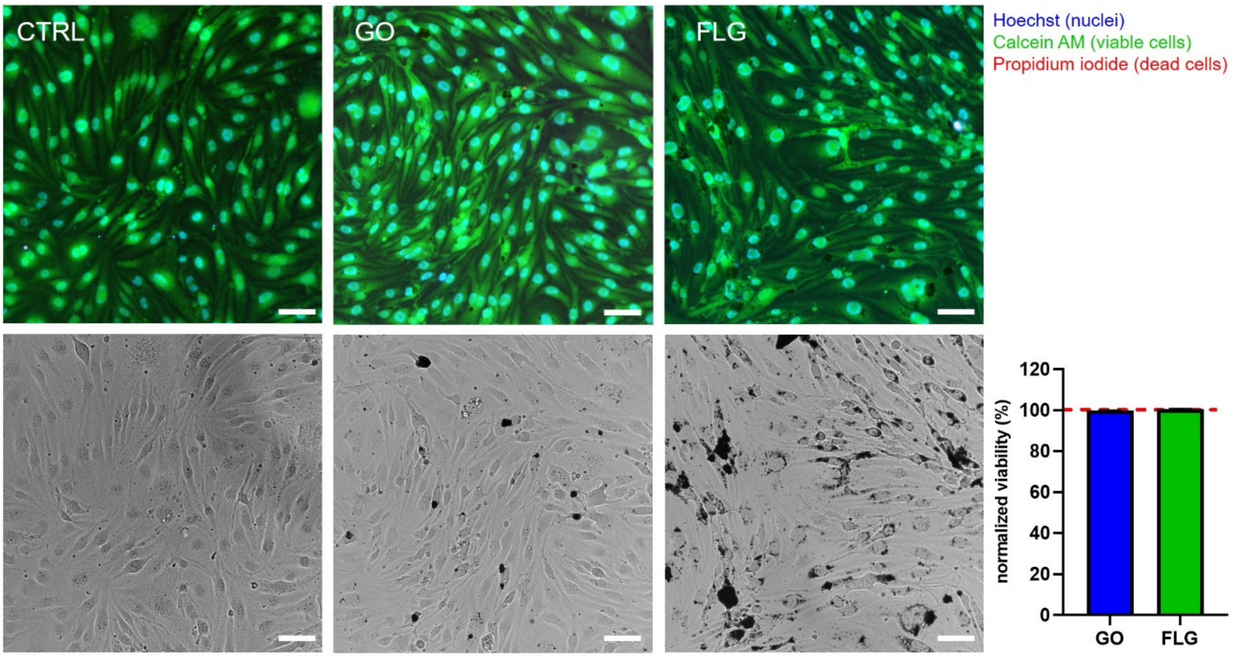

Figure S4. Cell viability for confluent bEnd.3 cells upon exposure to GO and FLG for 24 h. Representative fluorescence and bright field images of the bEnd.3 cell monolayer exposed to vehicle (Ctrl) or to 10 µg/ml of FLG or GO for 24 h. Cells were stained with Hoechst (nuclear stain for total cell number), calcein-AM (viable cells) and propidium iodide (dead cells). Scale bars: 50 µm. The bar plot on the bottom right shows the mean ± SEM) percent viability in the presence of either GO or FLG cells normalized over control cells. n=10 fields of view from 2 different independent cultures per condition.

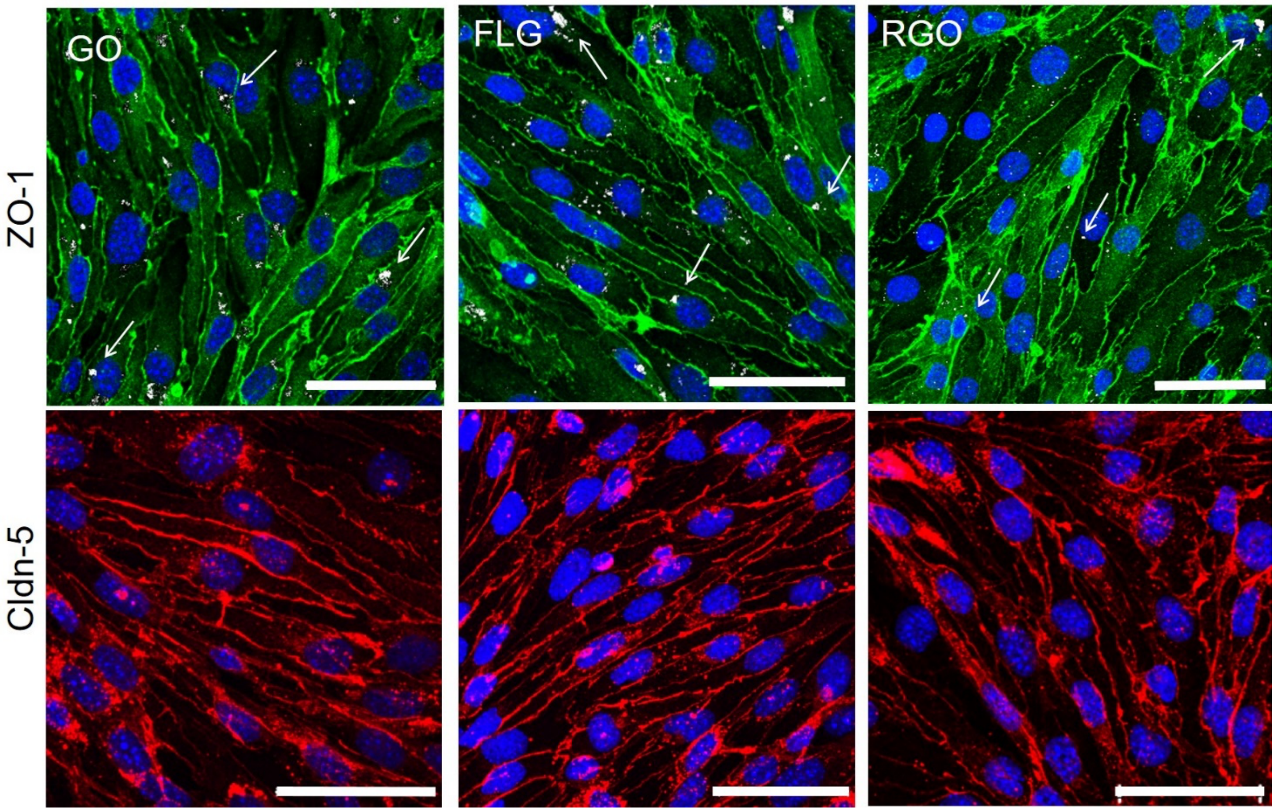

Figure S5. TJ expression in bEnd.3 cells after exposure to the three GRMs. Representative confocal images of immunofluorescence staining for ZO1 (green) and Claudin5 (red) in bEnd.3 monolayers at DIV9 seeded on glass coverslips after exposure to 10 µg/mL of either FLG or RGO for 48 h. The Hoechst dye was used for nuclei visualization (blue). Some GRM agglomerates (white arrows) are visible (reflection light mode). Scale bars: 50 µm.

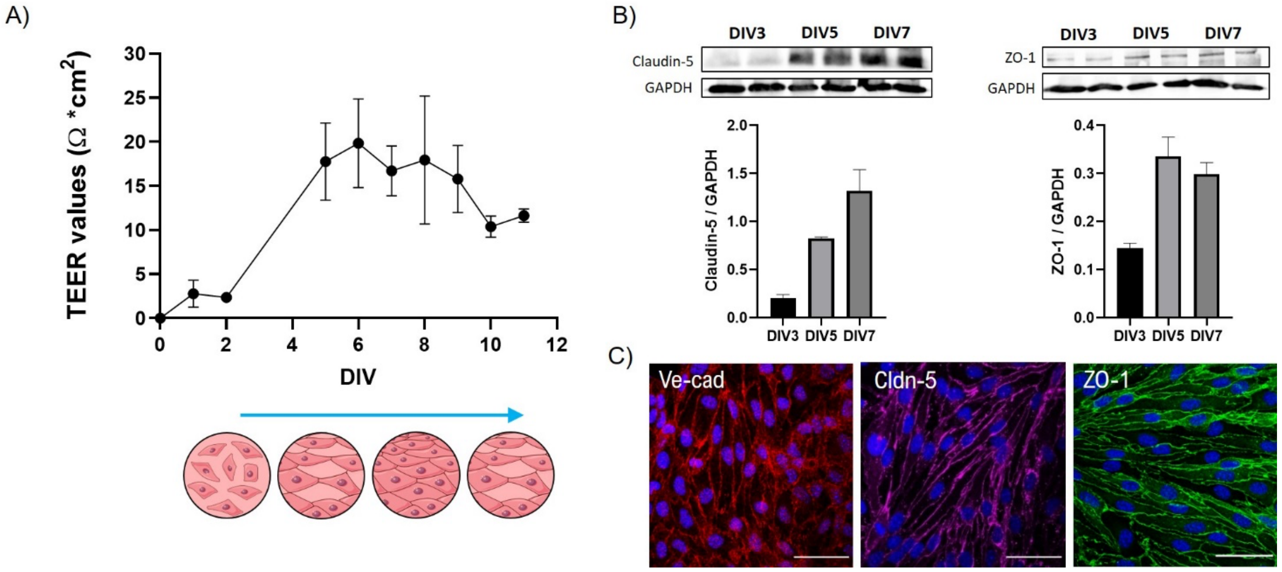

Figure S6. 2D murine BBB model characterization. A) Time-course of TEER values for a bEnd.3 cell layer cultured on a Transwell® membrane (pore size: 3 μm). TEER values increase overtime, indicating the tight junction formation, and stabilize around 15-20 Ω*cm^2 after 6 days. B) Western blot showing the increasing expression of Claudin-5 and Zonula Occludens 1 (ZO-1) in bEnd.3 cell lysate at DIV3, 5 and 7. In the graphs below, relative quantification is reported. C) Immunostaining of the main tight junction and adhesion proteins: Claudin-5, ZO-1 and Ve-cadherin (Ve-cad), on a bEnd.3 cell layer after 6 days of culture on a Transwell® membrane.

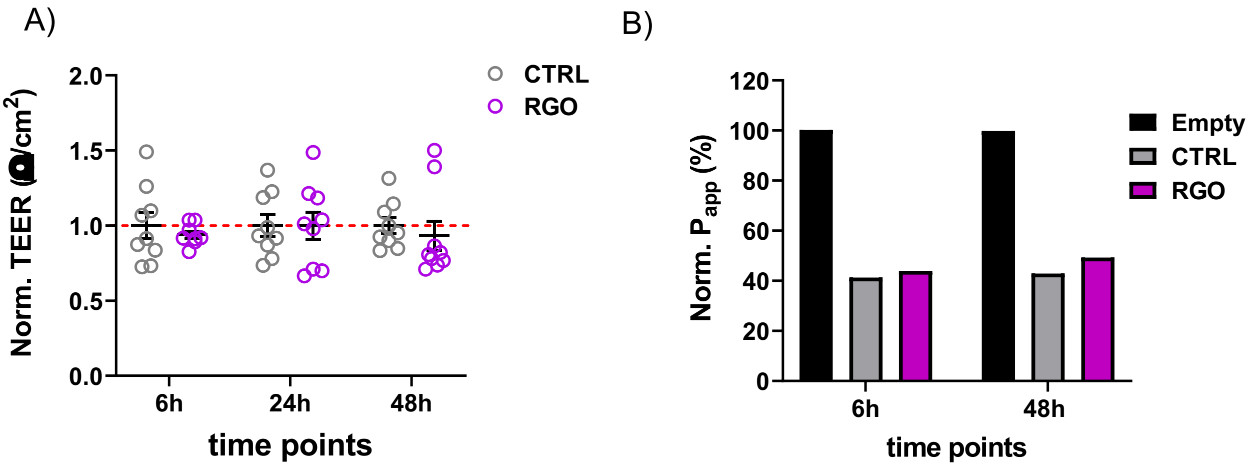

Figure S7. RGO does not affect BBB integrity and functionality. A) TEER values after exposure to 10 µg/mL of RGO for 6, 24 and 48 h. For each time point, values are normalized over the controls and are represented as means ± SEM, n=9. B) Apparent permeability (%) of FD4 after exposure to 10 µg/mL of RGO for 6 and 48 h. For each time point, values are normalized over control and are represented as means of triplicates, n=1.

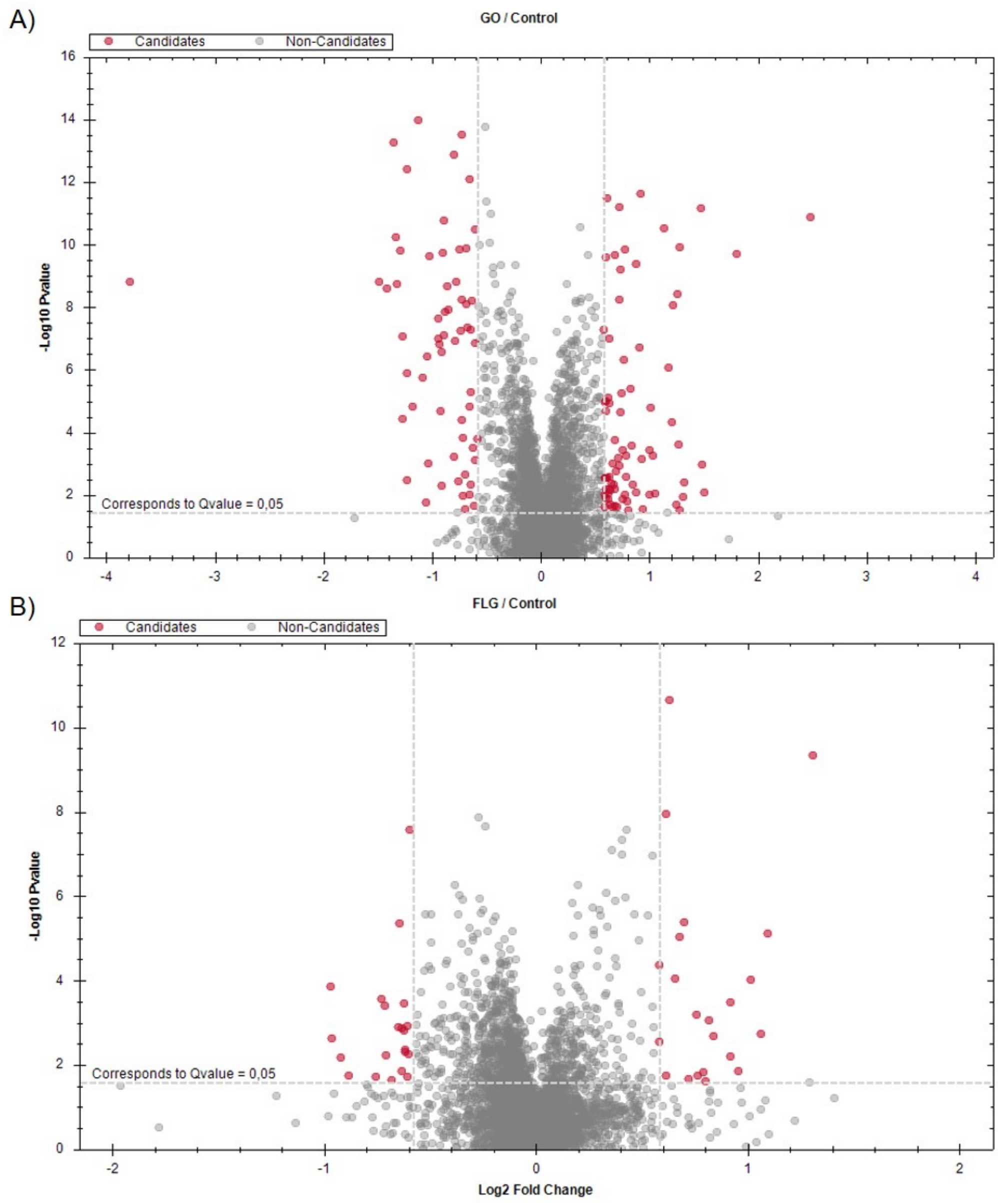

Figure S8. Proteomic analysis of bEnd.3 cells following exposure to GO and FLG. Volcano plot reporting the proteins whose expression is significantly altered ($p<0.05$) following exposure to either to GO (A) or FLG (B). The fold change threshold has been set to a fold change of 0.5.

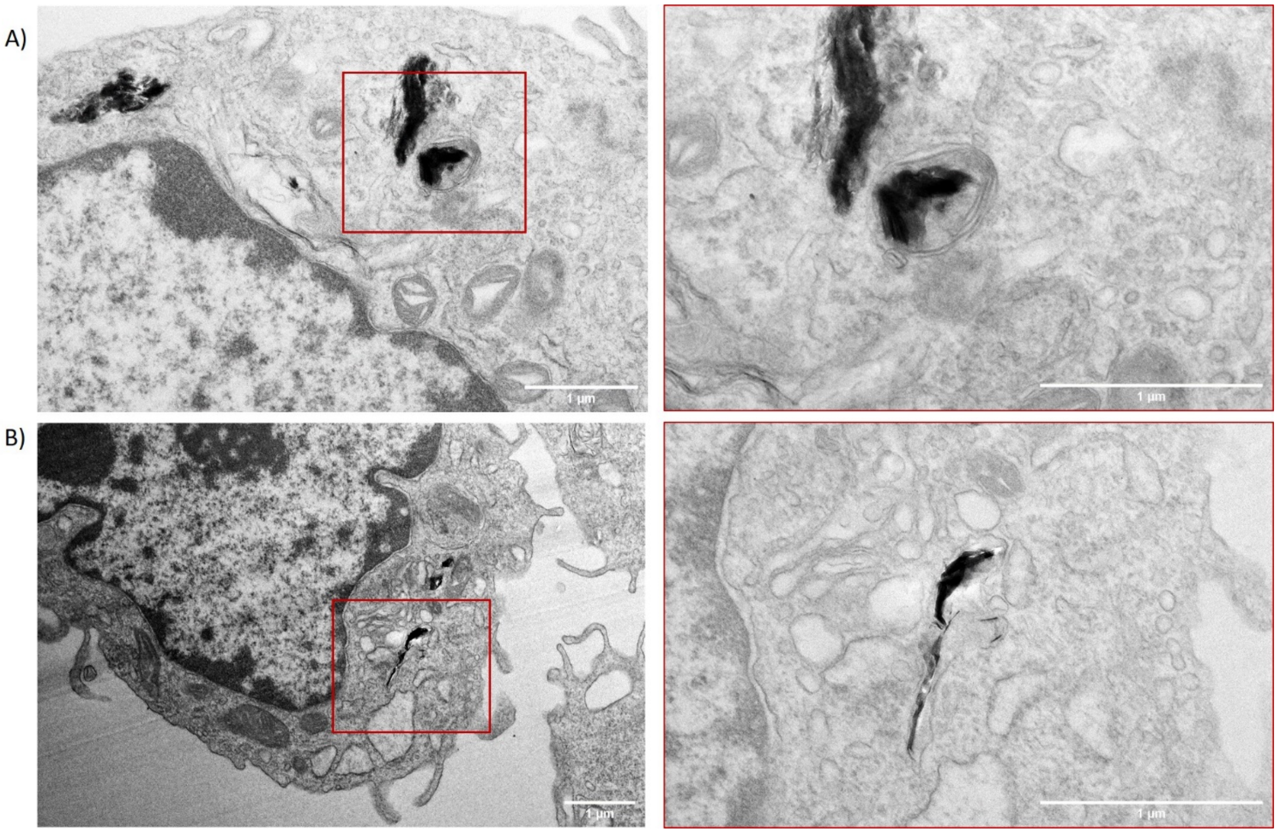

Figure S9. Electron microscopy images of bEnd.3 cells following exposure to GO and FLG. Additional TEM micrographs show internalization of either GO (A) or FLG (B) in bEnd.3 cells.

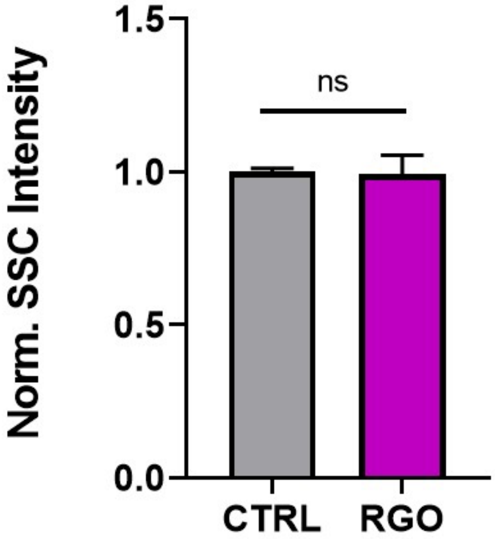

Figure S10. Uptake of RGO (10 µg/mL) by the bEnd.3 cell monolayer. Cells were exposed for 4 h. Data are expressed as means ± SEM, n = 3. Statistical analysis: unpaired Student's *t*-test.

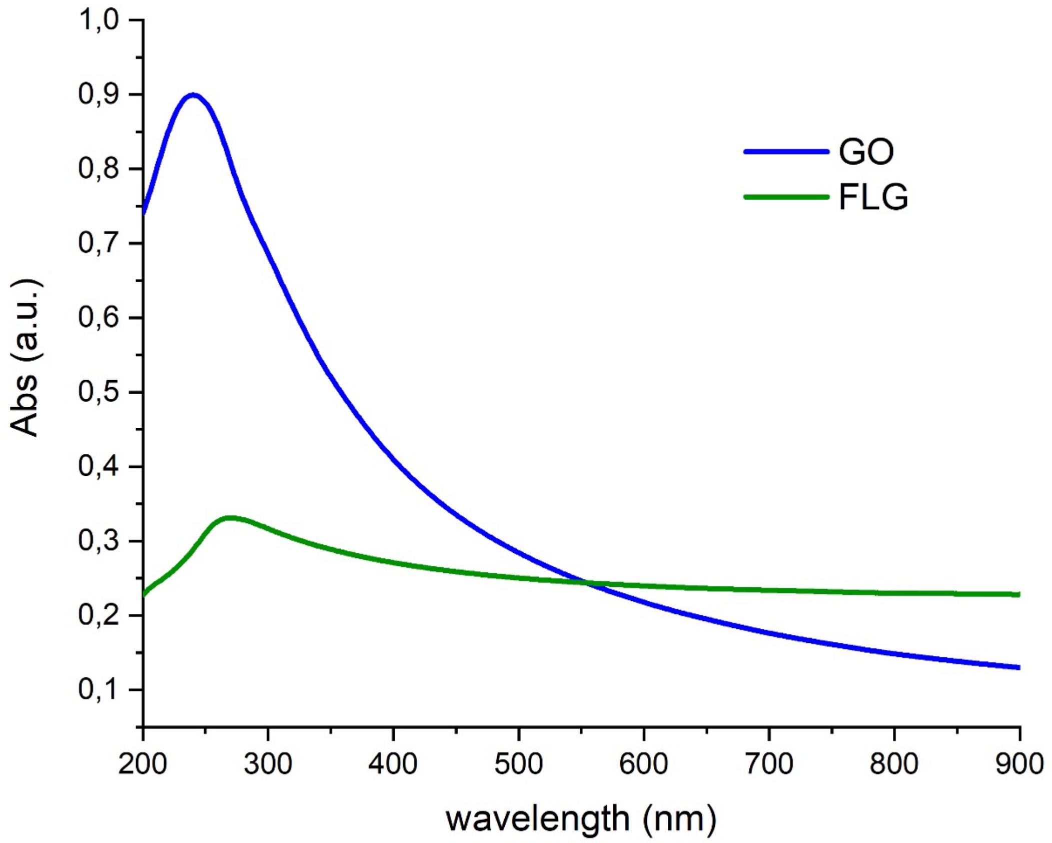

Figure S11. Absorption Spectra of GO and FLG. Distinct absorption profiles and scattering intensities are observed for GO (blue trace) and FLG (green trace), both at 10 µg/mL. While GO adsorbe more until 550 nm, scattering of FLG is more marked from 550 to 900 nm.

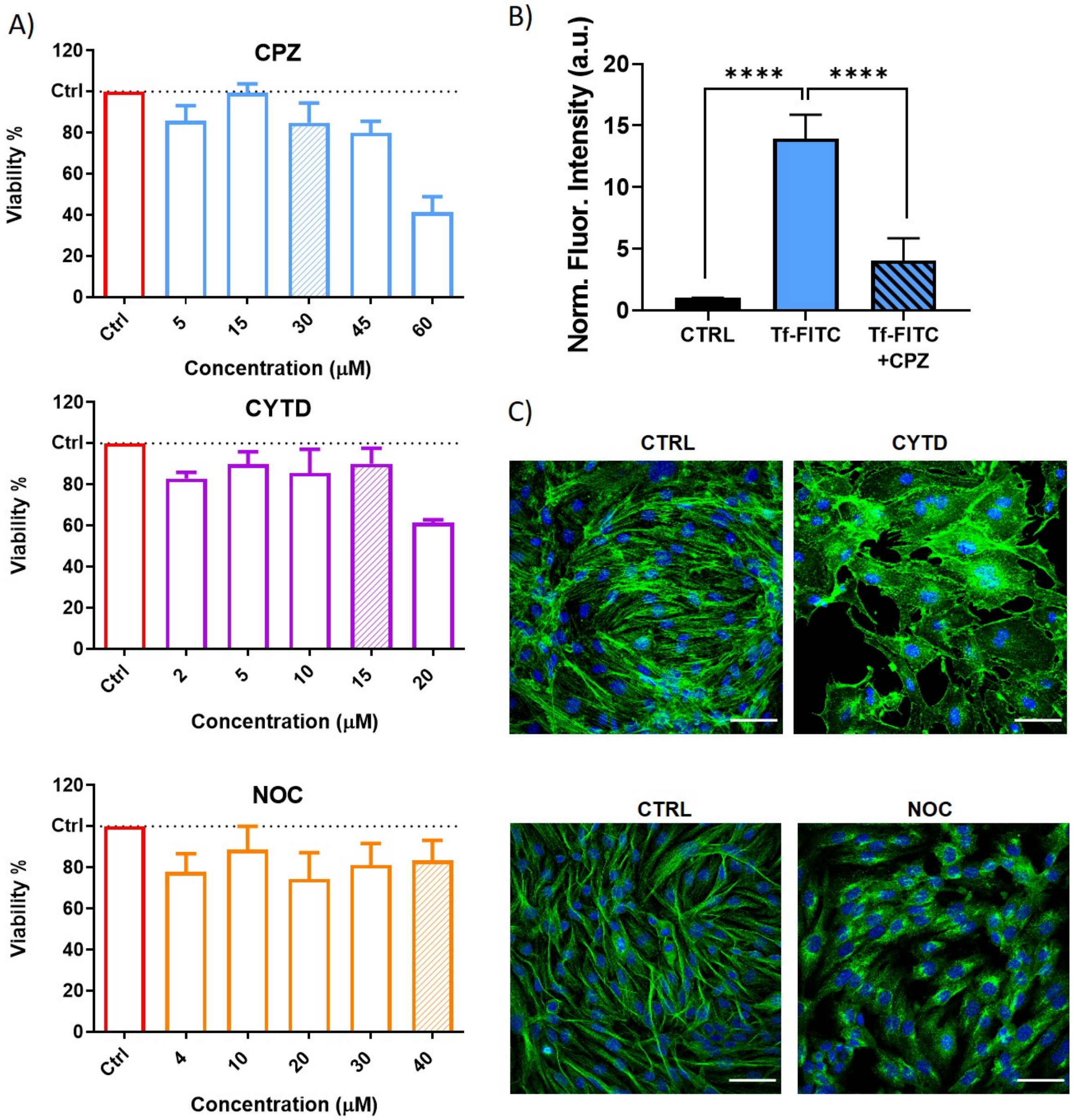

Figure S12. Optimization of the platform to analyze the cell internalization pathways. A) Viability of bEnd.3 exposed to increasing concentrations of inhibitors: chloropromazine hydrochloride (CPZ), cythochalasin D (CYTD) and nocodazole (NOC). B) Positive control for inhibition of clathrin-mediated endocytosis: upon cell treatment with 30 μM of CPZ for 2 h, the uptake of FITC-transferrin (as measured by flow cytometry) is significantly decreased. Values are represented as means ± SEM, n=3. Statistical analysis: ****= $p < 0.0001$, One-way ANOVA/Tukey's tests. C) Positive control for inhibition of phagocytosis (CYTD, top) and micropinocytosis (NOC, bottom). *Top:* Actin depolymerisation after treatment with 15 μM CYTD for 1 h, observed by confocal fluorescence microscopy of phalloidin-488 staining. *Bottom:* Microtubule disruption after treatment with 40 μM of NOC for 1 h, observed by confocal fluorescence microscopy of α-tubulin immune staining. Scale bars: 50 μm.

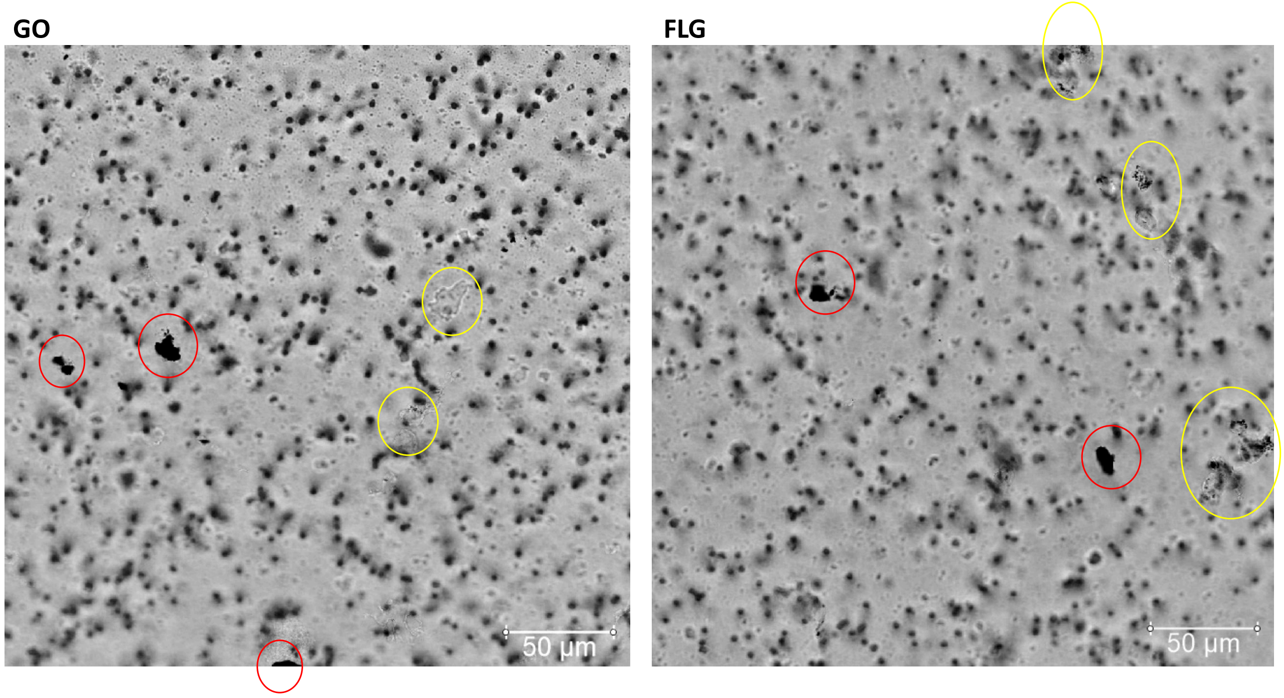

Figure S13. Bright field images of the apical Transwell® side after cell removal. Cells were exposed to GO and FLG (10 µg/mL for 24 h), washed multiple times with PBS and detached by trypsin. GRM aggregates (red circles) and cellular debris (yellow circles) are visible.

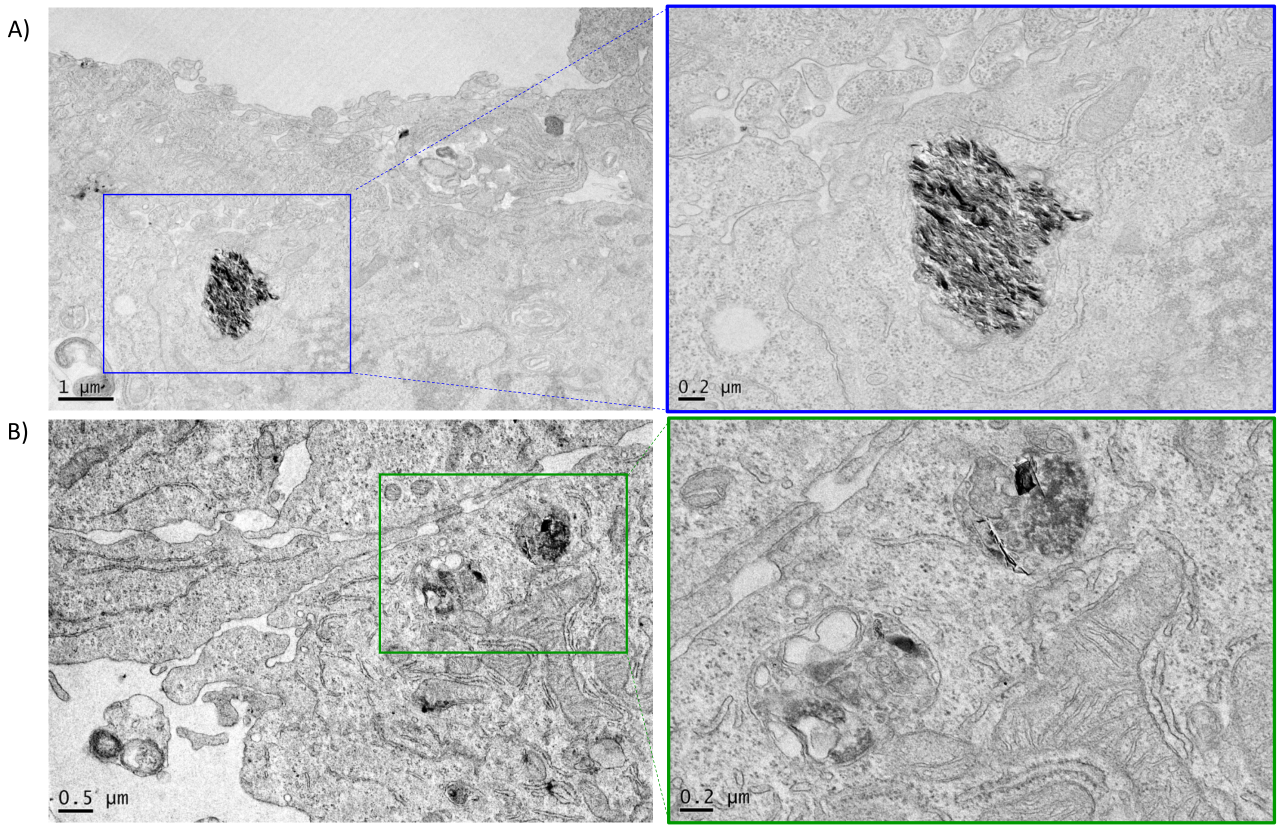

Figure S14. Electron microscopy of hMCA cells following exposure to GO and FLG. Additional TEM micrographs show internalization of either GO (A) or FLG (B) in hMCA cells.
